# Mechanism of RIP2 enhancing stemness of glioma cells induces temozolomide resistance

**DOI:** 10.1111/cns.13981

**Published:** 2022-10-02

**Authors:** Xiao‐liang Wang, Bao‐hua Jiao, Jian‐liang Wu, Jian‐kai Yang, Yu‐hua Hu, Kai Cui

**Affiliations:** ^1^ Department of Neurosurgery The Second Hospital of Hebei Medical University Shijiazhuang China; ^2^ Department of Neurosurgery The Fourth Hospital of Hebei Medcial University Shijiazhuang China

**Keywords:** drug resistance, glioma, NF‐κB, RIP2, stemness, temozolomide

## Abstract

**Aims:**

We aimed to investigate the role of receptor‐interacting protein 2 (RIP2) in regulation of stemness of glioma cells and chemotherapy resistance.

**Methods:**

Plasmid transfection was used to overexpress RIP2. Chemical inhibitors were used to inhibit RIP2 or NF‐κB activity. Cancer stemness of glioma cells was investigated by sphere formation assays, clone formation assays, and xenograft tumor formation assays. The expression of RIP2, p‐NF‐κB, IκBα, CD133, or SOX‐2 was detected by Western blotting and immunofluorescence. Apoptosis was detected by flow cytometry. Immunohistochemical staining was used to detect the expression of RIP2, CD133, and SOX‐2 in xenograft tumor tissue. The effect of the RIP2/NF‐κB pathway on temozolomide (TMZ) resistance was evaluated by xenograft tumor assay.

**Results:**

Transfection with RIP2 plasmid enhanced the sphere formation capability of U251 cells, clone formation capability, and xenograft tumor formation capability. RIP2 could mediate TMZ resistance by upregulating the expression of CD133 and SOX‐2 by activating the NF‐κB pathway. Both RIP2 inhibitor GSK583 and the NF‐κB inhibitor SC75741 could reverse the resistance of U251 cells to TMZ.

**Conclusion:**

RIP2 mediates TMZ resistance by regulating the maintenance of stemness in glioma cells through NF‐κB. Interventions targeting the RIP2/NF‐κB pathway may be a new strategy for TMZ‐resistant gliomas.

## Graphical abstract

RIP2 is involved in the induction of TMZ resistance in gliomas. The RIP2/NF‐κB signaling pathway induces enhanced stemness and plays an important role in TMZ resistance. The combined application of RIP2 or NF‐κB inhibitors and TMZ may be a new strategy for the treatment of RIP2‐related drug‐resistant gliomas.

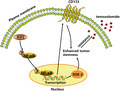

## 1 INTRODUCTION

Gliomas originate from the intrinsic constituent cells of the brain and are primary tumors of the central nervous system.^1^ The main adjuvant therapy after surgical treatment of glioma includes radiation therapy, chemotherapy, and molecular targeted therapy.^2^, ^3^ With the continuous improvement and updating of these treatment methods, the survival cycle and quality of life of glioma patients have been improved. However, most patients eventually die of disease progression caused by tumor recurrence, especially high‐grade gliomas, which are highly aggressive, have a high recurrence rate, and are extremely difficult to treat.^4^, ^5^

As a second‐generation oral alkylating agent, TMZ easily penetrates the blood–brain barrier and has a definite curative effect. It plays an important role in the treatment of gliomas.^6^ Inevitably, after a period of TMZ treatment, the treatment often fails due to development of acquired drug resistance, which is one of the major reasons for tumor progression.^7^ Therefore, the prevention of TMZ resistance is of significance for treating glioma. The causes of acquired drug resistance in gliomas are complex. Current studies have found that it is mainly related to the increased expression of ATP‐binding cassette transporters,^8^ enhanced stem cell characteristics,^9^ abnormal DNA damage repair systems,^10^ and antiapoptosis, up‐regulation of protein expression,^11^ and epithelial‐mesenchymal transition.^12^ There is increasing evidence that enhanced glioma stem cell properties are associated with TMZ resistance, but the signaling mechanisms are not fully clarified.

RIP2 is a kinase that is mainly involved in mediating immunity, inflammation, and apoptosis.^13^ In recent years, studies have found that RIP2 is expressed in various malignant tumor cells such as liver cancer,^14^ breast cancer,^15^ and bladder cancer^16^ and can mediate malignant biological behaviors such as tumorigenesis, cell migration and invasion, and metastasis. Increasing evidence shows that RIP2 plays an important role in mediating resistance of malignant tumor cells to drug therapy, such as resisting paclitaxel and ceramide‐induced apoptosis of breast cancer cells,^17^ reversing B‐lymphoproliferative disorders, and bortezomib resistance.^18^ The previous work of this study showed that RIP2 was involved in the resistance of gliomas to TMZ by inducing the expression of DNA repair enzyme MGMT.^19^ However, the relationship between RIP2‐mediated TMZ resistance and the stemness of glioma cells was still unclear, which was the focus of this study.

## 2 MATERIALS AND METHODS

### 2.1 Cell cultures

Lymphoma cells Raji were purchased from Nanjing Kebai Biotechnology Co. (Nanjing, China). Glioma cells U251 and U87 were purchased from Culture Collection of the Chinese Academy of Science (Shanghai, China). Glioma cells SW1783 and T98G were purchased from American Type Culture Collection (ATCC, Manassas, VA, USA). Raji and SW1783 cells were cultured in RPMI 1640 medium (Invitrogen, Carlsbad, CA, USA) containing 10% fetal bovine serum (FBS, Gibco‐BRL, Grand Island, NY, USA), and 1% penicillin–streptomycin (HyClone, Logan, UT, USA). T98G, U251, and U87 cells were cultured in DMEM high glucose medium (Invitrogen) containing 10% FBS and 1% penicillin–streptomycin. All cells were cultured in a 5% CO_2_, 37°C cell incubator.

### 2.2 CCK‐8 experiment

Briefly, cells were cultured in graded concentrations of TMZ (75, 150, 300, 600, 1200, 2400, and 4800 μM) for 72 h after necessary treatments. Five replicate wells were set up for each TMZ concentration. After TMZ treatment, 10 μl of CCK‐8 (Beyotime, Shanghai, China) solution was added to each well and incubated in an incubator for 3 h. The absorbance at 450 nm was read using an ELx800 microplate reader (BioTek, Winooski, VT, USA).

### 2.3 Transfection of RIP2 plasmid

The plasmid pEX‐3 containing the RIP2 gene (NM_003821.1) was constructed by GenePharma (Shanghai, China). Transfection was performed using GenJet™ Plus DNA In Vitro Transfection Reagent (Signagen, MD, USA, SL100499) according to the manufacturer's instructions.

### 2.4 Plate cloning experiments

The cells were seeded in 6‐well plates at 500 cells per well and cultured for 12–14 days. Cells were fixed with 4% paraformaldehyde (Beyotime), stained with 1% crystal violet staining solution (Beyotime), washed with water to remove the staining solution, and photographed with a camera.

### 2.5 Sphere formation assays

A serum‐free suspension medium was prepared using DMEM/F12 medium +20 ng/ml EGF + 20 ng/ml bFGF +2% B27. Cells were collected and seeded at 3000 cells/well in a low‐adsorption 12‐well plate for 10 consecutive days. Cell pellets were collected, trypsinized to prepare a single‐cell suspension, centrifuged to remove trypsin, and the suspension was prepared with serum‐free suspension medium. Per well 3000 cells were inoculated into a low‐adsorption 12‐well plate and cultured for 10 days to observe the cell spheroidization.

### 2.6 Western blotting

Cells were collected in centrifuge tubes and lysed with RIPA lysis buffer (Beyotime) to obtain total protein. The total protein concentration was measured in a BCA protein quantification kit (Beyotime), and a sample containing 3 μg/μl total protein was prepared. Proteins were separated by 10% SDS‐PAGE electrophoresis and transferred to 0.22 μm pore size PVDF membranes (Millipore, Billerica, MA, USA). PVDF membranes were blocked with 5% BSA (Beyotime) for 4 h and incubated with primary antibodies anti‐RIP2 (4142S, 1:1000), anti‐NF‐κB p65 (8242S, 1:2000), anti‐p‐NF‐κB p65 (3033S, 1:8000), anti‐IκBα (4812S, 1:1000), anti‐SOX‐2 (4900S, 1:1000), anti‐CD133 (64326S, 1:1000), and anti‐GAPDH (5174S, 1:1000) (Cell Signaling Technology, Danvers, MA, USA) overnight. Unbound primary antibody was washed away, and the PVDF membrane was labeled with horseradish peroxidase (HRP) goat anti‐mouse antibody (7076S, 1:3000) or goat anti‐rabbit antibody (7074S, 1:3000) (Cell Signaling Technology) and incubated for 1 h. Unbound secondary antibodies were washed away, and PVDF membranes were developed with SuperSignal West Femto Substrate (ThermoFisher, Waltham, MA, USA) and imaged using a GE AI600 system (GE, Chicago, IL, USA). Densitometric analysis of protein bands was performed using ImageQuant TL7.0 (GE).

### 2.7 Cell apoptosis analysis by flow cytometry

The cells to be tested were resuspended in binding buffer at 5 × 10^6^ cells/ml. 4 μl of Annexin V‐FITC (BioVision, China), and 4 μl of propidium iodide (Sigma) were added to 400 μl of cell suspension and incubated for 20 min at room temperature in the dark. Apoptosis was analyzed by flow cytometry (BD Bioscience, San Jose, CA, USA).

### 2.8 Immunofluorescence analysis

Cells were fixed with pre‐cooled methanol at 0–5°C for 20 min. After washing off the fixative, the cells were blocked with 3% BSA for 30 min at 15–25°C. The blocking solution was discarded, and cells were incubated with SOX‐2 (1:400) or CD133 (1:500) primary antibody. After washing away the unbound primary antibody, anti‐mouse IgG (H + L), F(ab’)2 Fragment (Alexa Fluor® 488 Conjugate) (1:500) and anti‐rabbit IgG (H + L), F(ab’)2 Fragment (Alexa Fluor® 594 Conjugate) (1:400) to incubate cells for 2 h, and then 0.5 μg/ml DAPI (with antifluorescence quencher) was added for 10 min. Cells were imaged under a CKX53 inverted fluorescence microscope (Olympus, Japan).

### 2.9 Animal experiments

Male nude mice (BALB/c‐nu) were purchased from GemPharmatech Co., Ltd. (Jiangsu, China) [License No. SCXK(SU) 2018–0008] and maintained on a 12‐hour day–night cycle with free food and water. The experiment included two parts. In the first part, U251 cells transfected with vector and RIP2 plasmids were inoculated into the back adjacent to the left axilla and the back adjacent to the right armpit of nude mice at 6 × 10^6^ cells each. Animals were sacrificed at 28 days, and tumors were weighed and photographed. In the second part of the experiment, U251 cells transfected with vector and RIP2 plasmids and untransfected U251 cells were inoculated in 6 × 10^6^ cells in the back of the right armpit of nude mice, and the tumors grew to a diameter of about 100 mm^3^ for grouping. The U251 tumor‐bearing mice transfected with RIP2 plasmid were tested alone and were divided into control group, TMZ group, SC75741 + TMZ group, and GSK583 + TMZ group. Transfected vector and untransfected U251 tumor‐bearing mice were used for experiments and were grouped into the control, vector, control+TMZ, and vector+TMZ groups. TMZ (20 mg/kg i.p.), SC75741 (5 mg/kg i.p.), and GSK583 (3 mg/kg p.o.) were administered once daily for 3 weeks. The control group was given an equal volume of normal saline. The tumor size was measured three times a week, and volume was calculated using the following formula: tumor volume (mm^3^) = (length × width^2^) × 1/2. Animals were anesthetized and sacrificed on day 25 after inoculation, harvested, weighed, and tumors were photographed. Relative tumor volume (RTV) was calculated according to the measurement results, and the calculation formula was: RTV = Vt/V1, where V1 is the tumor volume measured at the time of group administration (d1), and Vt is the tumor volume at each measurement. The relative tumor proliferation rate T/C (%) was calculated according to RTV, and the formula was as follows: T/C (%) = (TRTV / CRTV) × 100%, TRTV: RTV in the treatment group; CRTV: RTV in the control group. All tumors were fixed with 10% neutral buffered formalin for subsequent pathological examination. All animal experiments were carried out in accordance with the Declaration of Helsinki and the regulations for the care and use of laboratory animals of the State Food and Drug Administration of China. The experimental protocol was approved by the Animal Ethics Committee of the Second Hospital of Hebei Medical University.

### 2.10 Histological analysis

Fixed xenograft tissue was paraffin‐embedded and sectioned. Staining was performed with an immunohistochemical staining kit (ZSGB‐BIO Co., China). Briefly, paraffin sections were deparaffinized, hydrated, antigen retrieved, blocked endogenous peroxidase, blocked, and incubated with SOX‐2 (14,962 T, 1:300) or CD133 (1:200) primary antibody overnight (4°C). The next day, the primary antibody was washed off and incubated with biotin‐labeled goat anti‐mouse/rabbit IgG polymer and horseradishase‐labeled streptavidin working solution for 1 h each. The tissue sections were counterstained with hematoxylin after dripping with DAB chromogenic solution, dehydrated, mounted, microscopically imaged, and photographed. Integrated optical density (IOD) values of tissue sections in each group were measured by Image‐Pro Plus 6.0 software (Media Cybernetics, Rockville, MD, USA).

### 2.11 Statistical analysis

Statistical analysis was performed using SPSS 19.0 and GraphPad Prism 8.0 for Windows. All data conform to the normal distribution by Shapiro–Wilk test. Statistics are presented as mean ± standard deviation (*SD*). Statistical significance was calculated using one‐way analysis of variance (ANOVA) followed by Fisher's multiple comparison test. *P* < 0.05 indicated statistical significance.

## 3 RESULTS

### 3.1 RIP2 induces TMZ resistance in glioma cells

**FIGURE 1:**
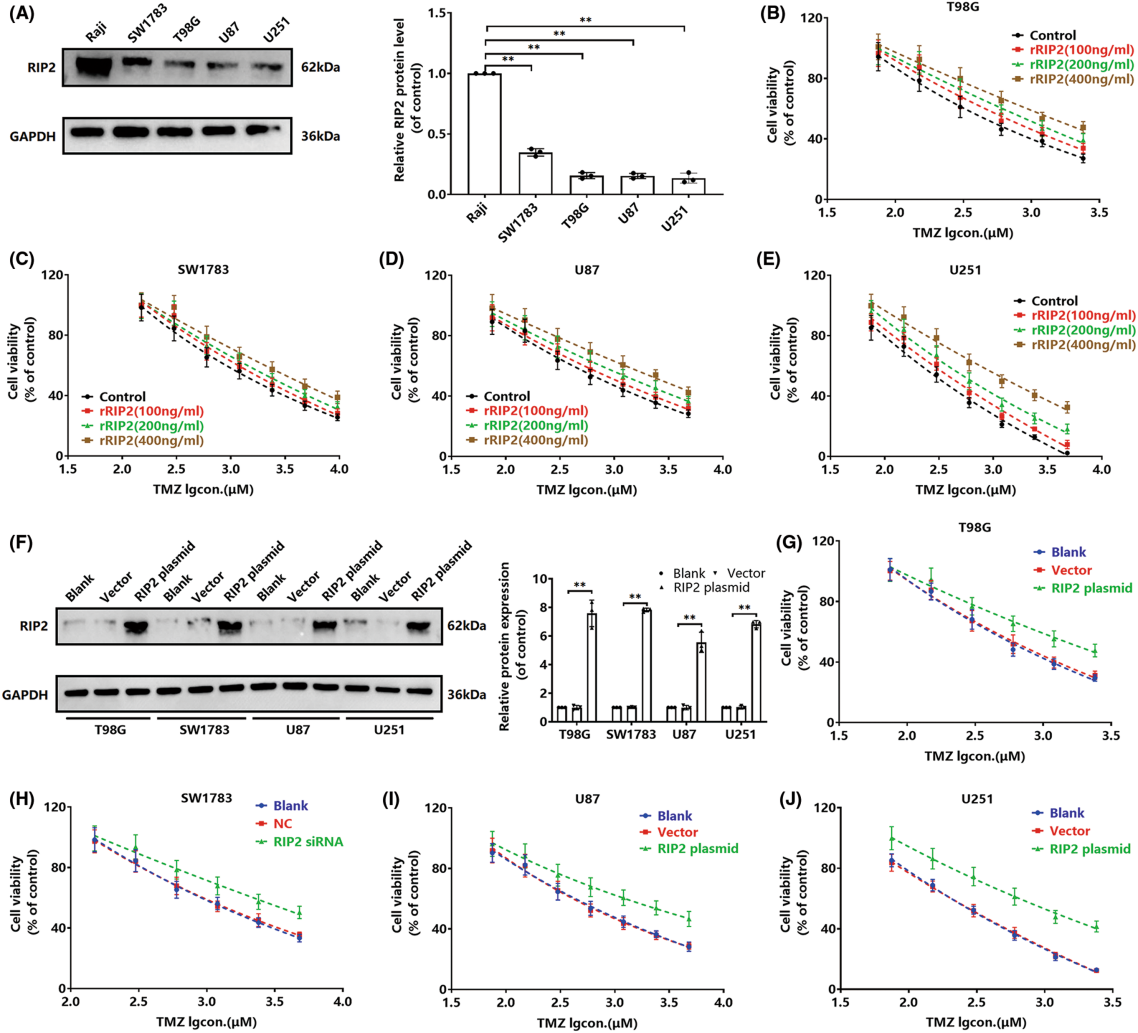
RIP2 induces glioma cells to resist TMZ. (A) Western blot assay to detect the expression of RIP2 in Raji, SW1783, T98G, U87, U251 cells. **P < 0.01. (B–E) Different glioma cells were treated with gradient concentrations of rRIP2 for 24 h and then TMZ was added (75, 150, 300, 600, 1200, 2400, and 4800 μM) for 72 h, and cell viability was detected by CCK‐8 assay. (F) T98G, SW1783, U87, and U251 cells were transfected with RIP2 plasmid and vector, respectively, and untransfected cells were used as negative controls. The expression of RIP2 was detected by Western blotting. **P < 0.01. (G–J) Different glioma cells transfected with RIP2 plasmid and vector were treated with TMZ (75, 150, 300, 600, 1200, 2400, and 4800 μM) for 72 h, and cell viability was measured by CCK‐8 assay. Normalization was performed with GAPDH as an internal reference.

To study the role of RIP2 in TMZ drug resistance of gliomas, we first used Raji cells with high RIP2 expression as a positive control to observe the endogenous expression of RIP2 in four gliomas (SW1783, T98G, U87, and U251). The results showed that all four gliomas could express RIP2, but the expression level was significantly weaker than that of Raji cells (Figure 1A). We further pretreated four types of cells with rRIP2 and observed the inhibitory effect of TMZ on cell proliferation. With increasing doses, rRIP2 significantly resisted the TMZ‐induced decrease in cell proliferation (Figure 1B–E). Among them, U251 cells were the most sensitive to TMZ. We then observed the sensitivity of cells to TMZ by exogenously upregulating RIP2 protein expression in four cell types. After RIP2 overexpression, all four cells were significantly less sensitive to TMZ (Figure 1F–J). Similarly, U251 cells remained the most sensitive to TMZ.

### 3.2 RIP2 enhances stemness of glioma cells

**FIGURE 2:**
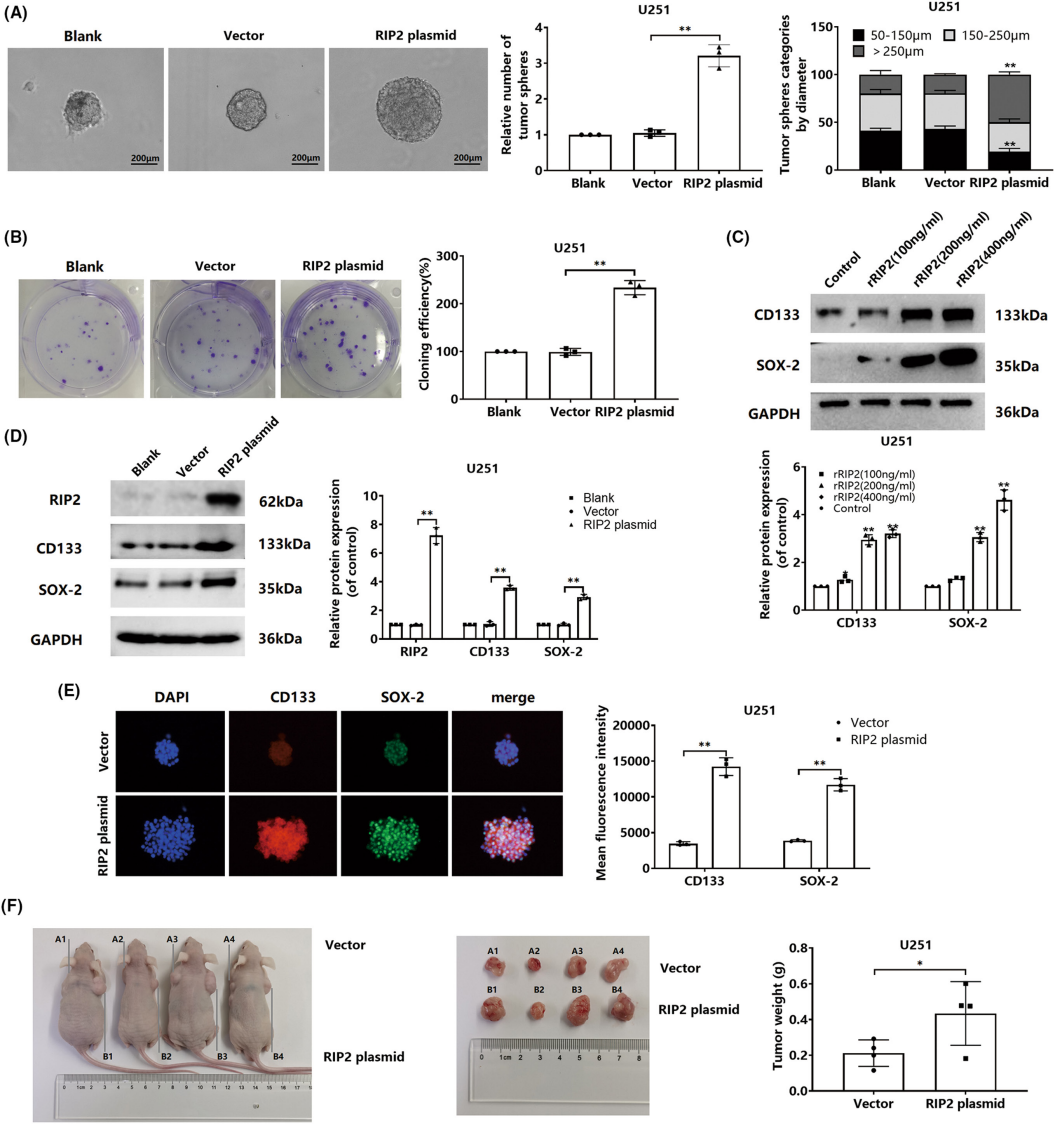
RIP2 enhances the stemness of glioma cells. (A) Using untransfected cells as negative control, a sphere formation assay of U251 was performed with cells transfected with RIP2 plasmid and vector. Compared with vector, **P < 0.01. (B) Plate cloning experiments were performed to test the clone formation ability of cells in blank, vector, and RIP2 plasmid groups. **P < 0.01. (C) U251 cells were treated with 100, 200, and 400 ng/mL rRIP2 for 24 h, and the expressions of CD133 and SOX‐2 were detected by Western blotting. Compared with control, **P<0.01. (D) Using untransfected cells as a negative control, U251 cells were transfected with RIP2 plasmid and vector, respectively, and the expressions of CD133 and SOX‐2 were detected by Western blotting. **P < 0.01. (E) The expression of CD133 and SOX‐2 in U251 cells of vector and RIP2 plasmid groups was detected by an immunofluorescence assay. **P < 0.01. (F) U251 cells transfected with vector and RIP2 plasmid were inoculated into the backs of the left and right armpits of nude mice, respectively, and sacrificed at 28 days to obtain pictures and measure the tumor weight. *P < 0.05

Considering that the effect of RIP2‐induced U251 cells on TMZ sensitization was the most obvious among the four types of cells, we took U251 cells as the research object for further study. Currently, it is unclear whether RIP2 is involved in the maintenance of stemness of glioma cells. We performed a sphere formation assay and found that overexpression of RIP2 enhanced sphere formation capability of U251 cell lines (Figure 2A). Then, plate cloning experiments found that overexpression of RIP2 significantly enhanced the colony forming ability of U251 cells. In vivo experiments showed that U251 xenografts overexpressing RIP2 had stronger colony forming ability (Figure 2F). These suggested that RIP2 might enhance the stemness of U251 cells. For further verification, we observed the expression changes in stem cell marker proteins (CD133 and SOX‐2) by Western blotting experiments. The results showed that both rRIP2 treatment and exogenous overexpression of RIP2 up‐regulated CD133 and SOX‐2 protein expression (Figure 2C and D). The immunofluorescence experiments also found that the expression of CD133 and SOX‐2 in U251 cells overexpressing RIP2 was significantly up‐regulated (Figure 2E).

### 3.3 RIP2 enhances stemness of glioma cells dependent on the NF‐κB pathway

**FIGURE 3:**
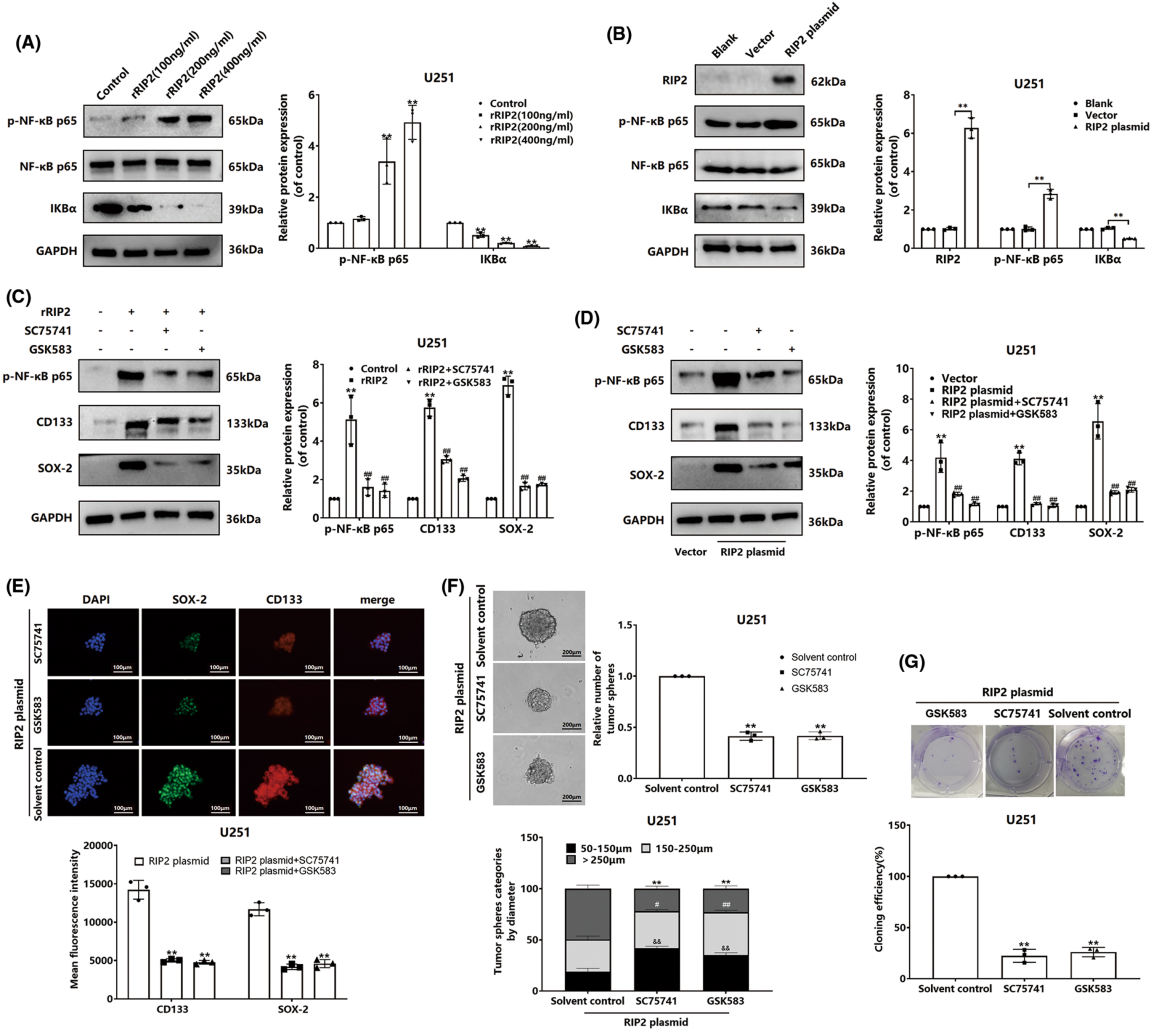
RIP2 enhances the stemness of glioma cells dependent on the NF‐κB pathway. (A) U251 cells were treated with 100, 200, and 400 ng/mL rRIP2 for 24 h, and the expressions of p‐NF‐κB and IκBα were detected by Western blotting. Compared with control, **P<0.01. (B) Using untransfected cells as negative control, U251 cells were transfected with RIP2 plasmid and vector, respectively, and the expression of p‐NF‐κB and IκBα was detected by Western blotting. Compared with Vector, **P < 0.01. (C) U251 cells were pretreated with a chemical inhibitor of RIP2 (GSK583, 1 μM) or a chemical inhibitor of NF‐κB (SC75741, 1 μM) and then stimulated with rRIP2. Western blotting was used to detect p‐NF‐κB, CD133 and SOX‐2 expression. **P < 0.01 compared with Control. ^##^ *P* < 0.01 compared with rRIP2 group. (D) U251 cells overexpressing RIP2 were pretreated with GSK583 or SC75741, and the expressions of p‐NF‐κB, CD133, and SOX‐2 were detected by western blotting. **P < 0.01 compared with the Vector group. ^##^ *P* < 0.01 compared to the RIP2 plasmid group. (E) U251 cells overexpressing RIP2 were pretreated with GSK583 or SC75741, and the expressions of CD133 and SOX‐2 were detected by immunofluorescence assay. **P < 0.01 compared with the RIP2 plasmid group. (F) Sphere formation assay for detection of RIP2‐overexpressing U251 cells pretreated with GSK583 or SC75741. **P < 0.01, ^#^ *P* < 0.05, ^##^ *P* < 0.01, ^&&^ *P* < 0.01, compared with the solvent control group. (G) Clone formation assays to detect RIP2‐overexpressing U251 cells pretreated with GSK583 or SC75741. **P < 0.01 compared with the solvent control.

To observe whether NF‐κB pathway is involved in RIP2‐mediated maintenance of stemness in glioma cells, we first detected the expression of NF‐κB pathway‐related proteins. Western blot results showed that rRIP2 up‐regulated p‐NF‐κB p65 expression and down‐regulated IκBα expression in U251 cells (Figure 3A). At the same time, similar changes in expression also occurred in p‐NF‐κB p65 and IκBα in U251 cells transfected with RIP2 plasmid (Figure 3B). Subsequently, we pretreated U251 cells with RIP2 and NF‐κB chemical inhibitors (GSK583 and SC75741) and then observed the effect of rRIP2 treatment or RIP2 overexpression on the expression of stem cell marker proteins. The results showed that either GSK583 or SC75741 pretreatment could inhibit the up‐regulation of CD133 and SOX‐2 protein expression induced by rRIP2 or RIP2 overexpression (Figure 3C,D). Immunofluorescence staining showed that either GSK583 or SC75741 pretreatment could inhibit the up‐regulation of CD133 and SOX‐2 protein expression induced by RIP2 overexpression (Figure 3E). The sphere formation assay and clone formation experiments showed that overexpression of RIP2 enhanced the sphere formation capability and clone formation capability of U251 cell lines (Figure 3F,G).

### 3.4 Inhibition of RIP2/NF‐κB signaling pathway inhibits RIP2‐induced TMZ resistance in glioma cells

**FIGURE 4:**
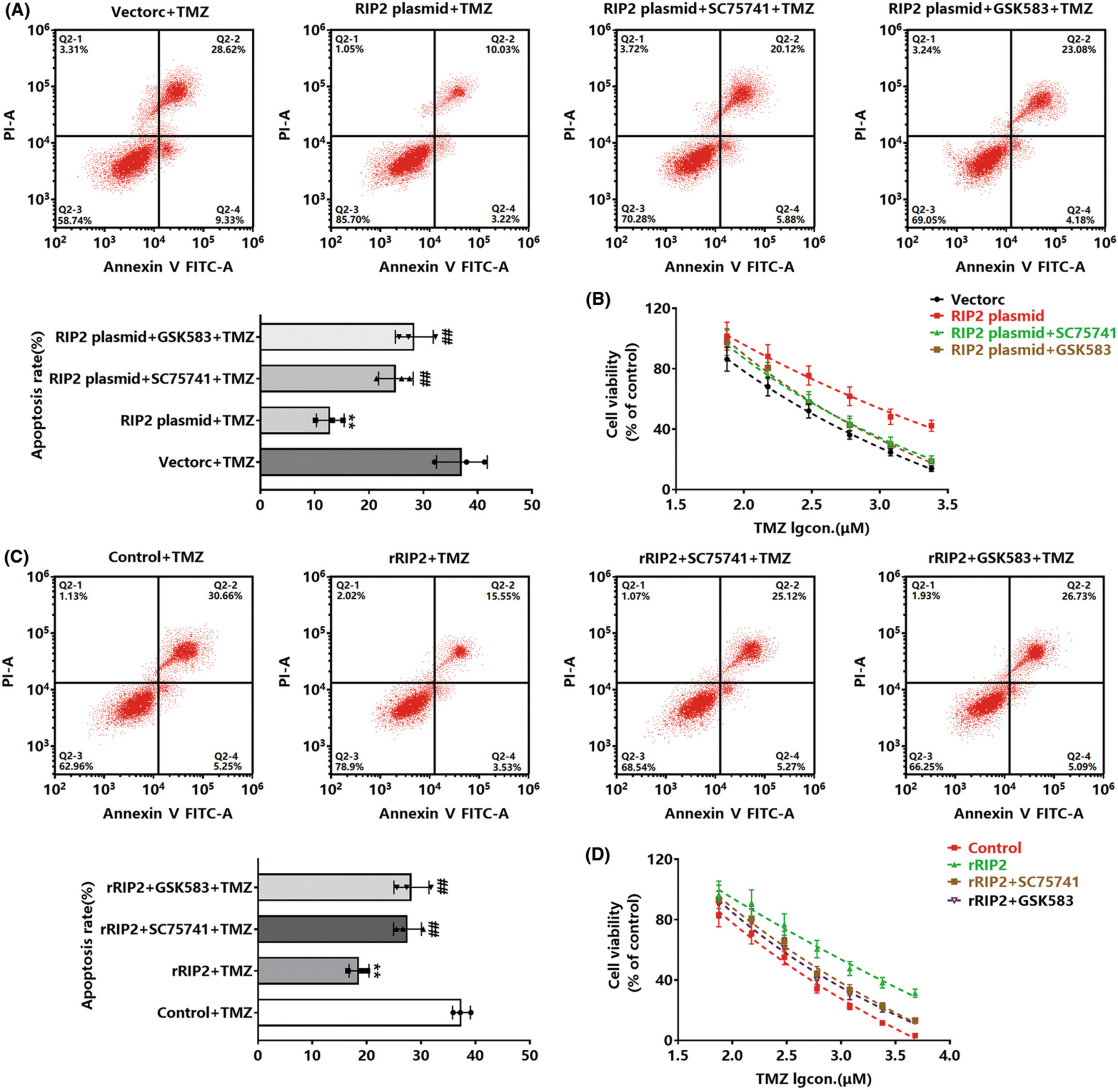
Inhibition of RIP2/NF‐κB signaling pathway inhibits RIP2‐induced TMZ resistance in glioma cells. (A) Vector, RIP2 plasmid, RIP2 plasmid+SC75741, and RIP2 plasmid+GSK583 cells were treated with TMZ for 72 h, and cell apoptosis was detected by flow cytometry. **P < 0.01 compared with vector+TMZ group. ^##^ *P* < 0.01 compared with the RIP2 plasmid+TMZ group. (B) Vector, RIP2 plasmid, RIP2 plasmid+SC75741, and RIP2 plasmid+GSK583 groups were treated with gradient concentrations of TMZ for 72 h, and cell viability was detected by CCK‐8 experiment. (C) Cells in control, rRIP2, rRIP2 + SC75741, and rRIP2 + GSK583 groups were treated with TMZ for 72 h, and cell apoptosis in each group was detected by flow cytometry. **P < 0.01 compared with the control+TMZ group. ^##^ *P* < 0.01 compared with the rRIP2 + TMZ group. (D) Cells in control, rRIP2, rRIP2 + SC75741, and rRIP2 + GSK583 groups were treated with gradient concentrations of TMZ for 72 h, and cell viability was detected by CCK‐8 assay.

Our study revealed that RIP2 enhanced glioma cell stemness through the NF‐κB pathway and induced cellular TMZ resistance. To confirm that RIP2 induces TMZ resistance in glioma cells dependent on NF‐κB, we used flow cytometry to observe the effect of TMZ on apoptosis of RIP2‐overexpressing U251 cells pretreated with GSK583 or SC75741. Transfection of the RIP2 plasmid inhibited TMZ‐induced apoptosis in U251 cells, while either GSK583 or SC75741 reversed the effect of RIP2 (Figure 4A). Detecting the effect of TMZ on the proliferation of cells in each group found that GSK583 or SC75741 could enhance the cytotoxic effect of TMZ on RIP2‐overexpressing U251 cells (Figure 4B). Our further validation with rRIP2 also found similar results to the above (Figure 4C,D).

### 3.5 RIP2 enhances the stemness of glioma xenografts through the NF‐κB pathway

**FIGURE 5:**
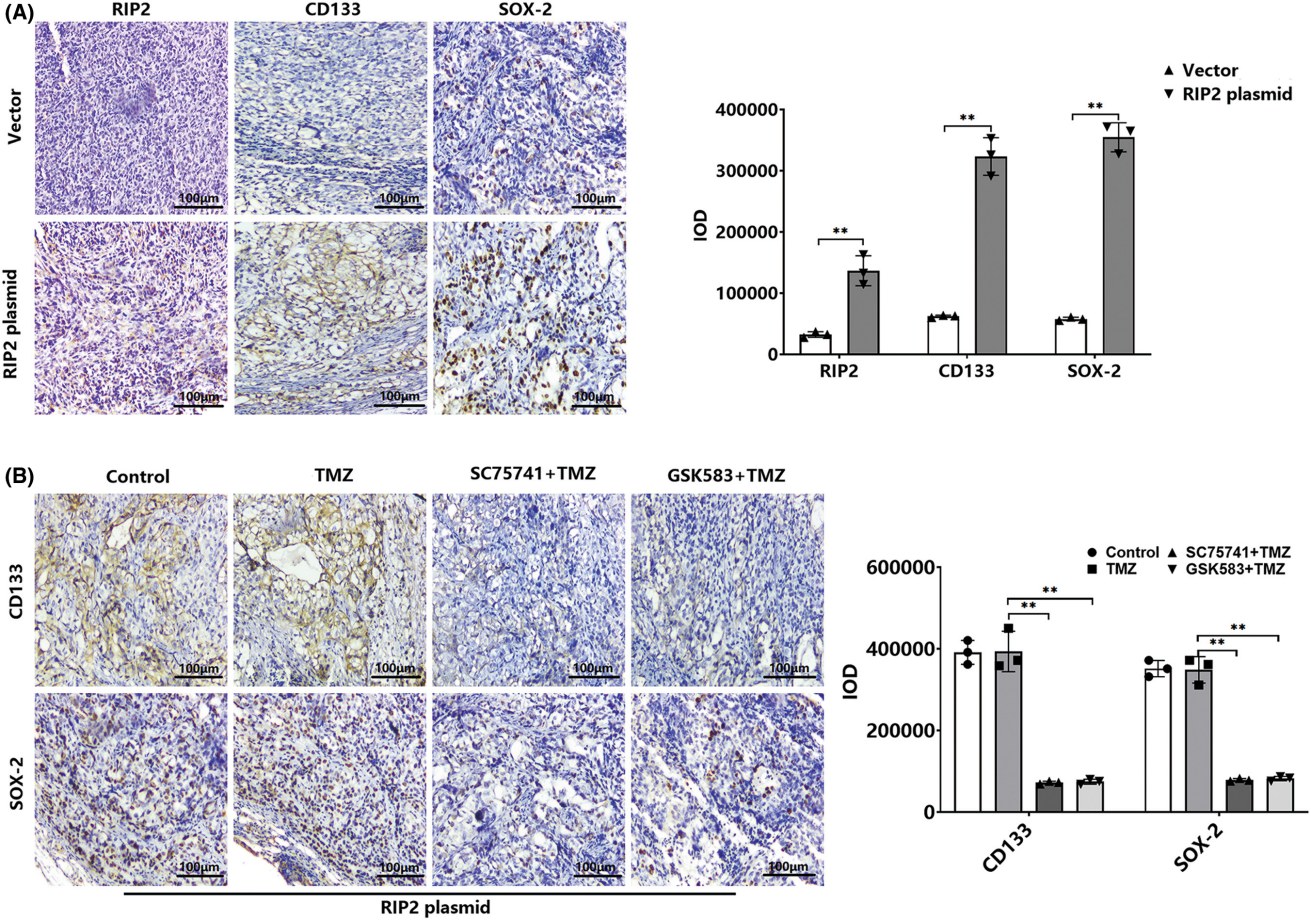
RIP2 enhances the stemness of glioma xenografts through the NF‐κB pathway. (A) Representative image of U251 tumor sections transfected with vector and RIP2 plasmid were stained with RIP2, CD133, and SOX‐2 antibody (×200). Scale bar: 100 μm. **P < 0.01. (B) Representative images of RIP2‐overexpressing U251 tumor sections in TMZ, SC75741 + TMZ, and GSK583 + TMZ groups stained with CD133 and SOX‐2 antibody (200×). Scale bar: 100 μm. **P < 0.01

We had found from in vitro studies that RIP2 enhances the expression of glioma stem cell marker proteins CD133 and SOX‐2 through the NF‐κB pathway. To further confirm the biological role of RIP2 in vivo, we used a xenograft tumor model. First, we observed the expression of CD133 and SOX‐2 in U251 cell xenografts transfected with RIP2 plasmid. Immunohistochemical staining showed that the expression of CD133 and SOX‐2 in U251 tumor tissue transfected with RIP2 plasmid was significantly up‐regulated (Figure 5A). Subsequently, we treated RIP2‐overexpressing U251 cells with SC75741 and GSK583 and found that both could down‐regulate CD133 and SOX‐2 protein expression (Figure 5B).

### 3.6 Inhibition of RIP2/NF‐κB can inhibit RIP2‐overexpressing glioma xenografts resistant to TMZ

**FIGURE 6:**
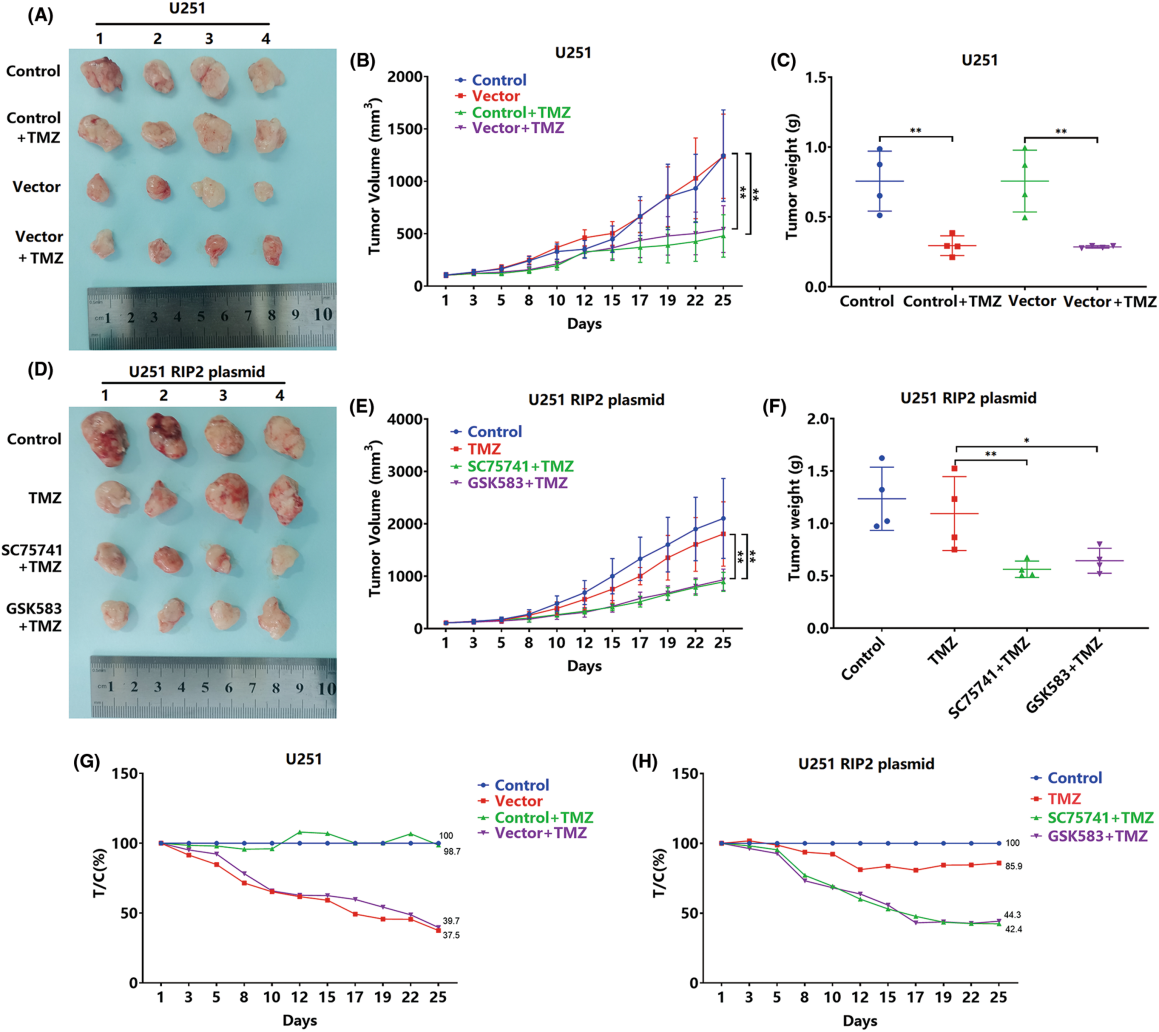
Inhibition of RIP2/NF‐κB can inhibit RIP2‐overexpressing glioma xenografts resistant to TMZ. (A) Qualified normal U251 cells and vector‐transfected U251 cell tumor‐bearing mice were treated with TMZ for 25 days. Xenograft tumors were excised and photographed. Changes in tumor volume of tumor‐bearing mice in control, vector, control+TMZ, and vector+TMZ groups from one to 25 days. ** *P* < 0.01. (C, F) Xenograft tumor weights in each group at 25 days. **P < 0.01. (D) Qualified U251 cell tumor‐bearing mice transfected with RIP2 plasmid were treated with TMZ, SC75741 + TMZ, and GSK583 + TMZ for 25 days. Xenograft tumors were excised and photographed. (E) Changes in tumor volume of tumor‐bearing mice in control, TMZ, SC75741 + TMZ, and GSK583 + TMZ groups from one to 25 days. **P < 0.01. (G, H) Changes in relative tumor proliferation rate (T/C) of xenograft tumors in each group.

We finally observed RIP2/NF‐κB‐induced TMZ resistance in a xenograft tumor model. First, we treated normal U251 cells and empty vector‐transfected U251 cell tumor‐bearing mice with TMZ. The results showed that the sensitivity of the two transplanted tumors to TMZ was strong and that there was no significant difference, with T/C ratios of 37.5% and 39.7%, respectively (Figure 6A–C,G). Subsequently, we constructed a RIP2‐overexpressing U251 cell xenograft tumor model. The xenograft was less sensitive to TMZ, with a T/C of only 85.9%. After treatment with SC75741 or GSK583, TMZ had better tumor‐inhibitory effect in this model, with T/C ratios of 42.4% and 44.3%, respectively (Figure 6D–F,H).

## 4 DISCUSSION

Multidisciplinary comprehensive treatment has gradually emerged with the development of medical and health services. The treatment of brain gliomas (especially glioblastomas), however, is still not optimal.^20^, ^21^ The clinical application of the alkylating agent TMZ shows promise for the treatment of malignant gliomas; however, acquired drug resistance eventually leads to treatment failure.^7^ RIP2 kinase has been shown to play a role in acquired drug resistance.^17^, ^18^, ^19^ This study found that four glioma cell lines. (SW1783, T98G, U87, and U251) stimulated by rRIP2 demonstrated enhanced resistance to TMZ. We found similar results after the four cell lines. Transfected with RIP2 plasmids exogenously overexpressed RIP2. This suggests that RIP2 plays a role in promoting glioma resistance to TMZ. We selected U251 cells for further study because RIP2 induced the strongest effect against TMZ in U251 cells among the four cell types we used.

Cancer stem cells are a significant cause of cancer occurrence, development, drug resistance, and recurrence due to their capacity for self‐renewal, limitless proliferation, and treatment resistance.^22^, ^23^, ^24^ In recent years, research has focused on regulating stem cell characteristics and the mechanism of drug resistance in tumor cells. Glioma stem cells are also characterized by self‐renewal and limitless proliferation, which is one of the reasons why gliomas are resistant to radiotherapy and chemotherapy.^25^ Effectively inhibiting the formation of stemness may be a new strategy in treating acquired resistance. Our studies found that, in vitro, U251 cells transfected with RIP2 plasmids exhibited enhanced stem cell sphere and colony formation. Similarly, in vivo, overexpression of RIP2 in U251 cells increased tumorigenicity. This indicates that RIP2 may induce and maintain the stemness of U251 cells.

Furthermore, we found that CD133 and SOX‐2 were up‐regulated in U251 cells treated with rRIP2 or transfected with RIP2 plasmids. We found that CD133 and SOX‐2 were up‐regulated in U251 cells and xenograft tumor tissue treated with rRIP2 or transfected with RIP2 plasmids. However, the RIP2 inhibitor GSK583 inhibited the up‐regulation effects. We also found that GSK583 could reverse TMZ resistance induced by RIP2 overexpression. CD133, a 5‐transmembrane glycoprotein, is an important marker of various cancer stem (including glioma) cells,^26^, ^27^ and is associated with chemoresistance of glioma cells. Studies have shown that the resistance of hypoxia‐induced glioblastoma to cisplatin is related to CD133, and silencing CD133 expression can reverse the induction effect of hypoxia.^28^ SOX‐2 plays a vital role in the early development of mammalian organs and is one of the necessary transcription factors for regulating mammalian embryonic development. SOX‐2 is also a common marker in cancer stem cells, which may be involved in the chemoresistance of glioma cells. Studies have shown that SOX2 plays a crucial role in regulating the chemoresistance of CD133(+) glioblastoma.^29^ It has also been reported that long non‐coding RNA PVT1 induces up‐regulation of SOX2 expression through the miR‐365/ELF4 axis, thereby promoting the maintenance of stemness and TMZ resistance in glioma cells.^30^ Knockdown of PVT1 and overexpression of miR‐365 has been found to inhibit stemness and TMZ resistance of glioma cells.^30^ This suggests that RIP2 may maintain the stemness of U251 cells by up‐regulating CD133 and SOX‐2, thereby resisting TMZ.

**FIGURE 7:**
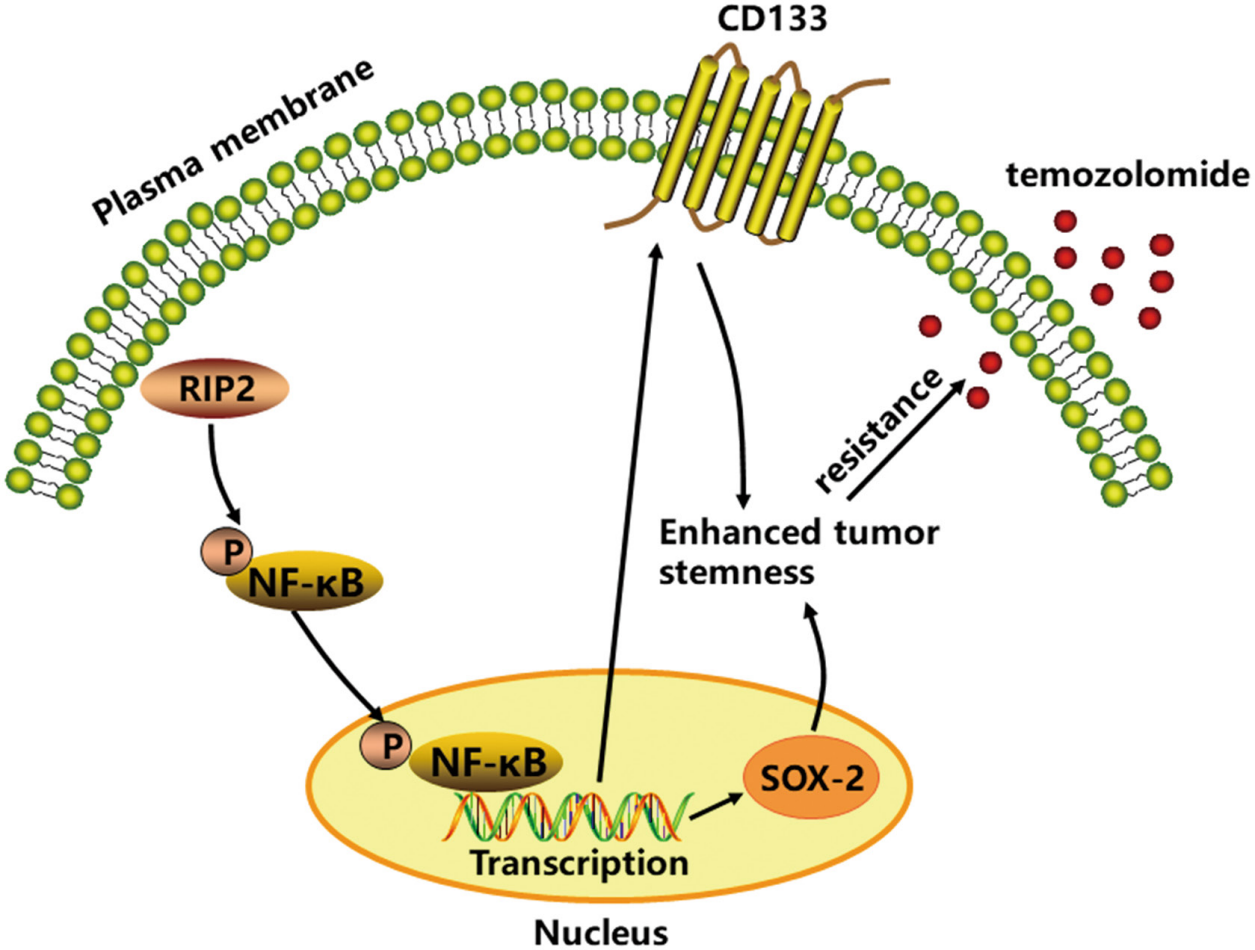
Schematic model of the RIP2 mediates TMZ resistance by regulating the maintenance of stemness in glioma cells through NF‐κB signaling pathway.

The NF‐κB signaling pathway is the cascade that initiates nuclear transcription, which plays a key role in the cellular inflammatory, immune, and emergency responses.^31^, ^32^ Studies have shown that RIP2 promotes triple‐negative breast cancer cell migration and invasion by activating NF‐κB and c‐Jun N‐terminal kinase pathways.^15^ RIP2 can activate the NF‐κB signaling pathway to resist paclitaxel‐ and ceramide‐induced apoptosis in breast cancer cells.^17^ RIP2 also interacts with PAX5 to promote resistance of B‐lymphoproliferative disorders to bortezomib by activating NF‐κB.^18^ In glioma cells, RIP2 promotes cell growth by regulating TRAF3 and activating NF‐κB and p38 signaling pathways.^33^ Previously, we found that RIP2 regulates MGMT expression through the NF‐κB signaling pathway, thereby mediating the resistance of glioma cells to TMZ.^19^ This suggests that NF‐κB is a downstream signaling target of RIP2, and activation of NF‐κB may be involved in RIP2‐induced enhancement of stemness of glioma cells. Therefore, we observed the activation state of the NF‐κB signaling pathway and found that rRIP2 or transfection of the RIP2 plasmids activated the NF‐κB signaling pathway. This is consistent with the published literature^15^, ^17^, ^18^, ^19^, ^33^ Studies have shown that activating stem cell developmental pathways such as Wnt,^34^ Notch,^35^ and Hedgehog^36^ can confer stem cell characteristics to cancer cells. The NF‐κB signaling pathway is, however, also involved in maintaining cancer cell stemness. For example, NF‐κB maintains the stemness of colon cancer cells by down‐regulating miR‐195‐5p/497‐5p and up‐regulating MCM2.^37^ To investigate whether NF‐κB is involved in mediating RIP2‐induced stemness of U251 cells, we observed the intervention effect of the NF‐κB chemical inhibitor SC75741. In both in vitro and in vivo studies, SC75741 treatment inhibited RIP2‐induced upregulation of CD133 and SOX‐2 expression. It is suggested that RIP2 maintains the stemness of U251 cells through the NF‐κB signaling pathway. In cell and xenograft studies, we found that SC75741 treatment inhibited TMZ resistance induced by RIP2 overexpression. These findings suggest that RIP2 can induce U251 cells to resist TMZ, and this effect is related to the activation of the NF‐κB signaling pathway and maintenance of stemness of U251 cells (Figure 7).

A retrospective analysis of many studies found that stem cell therapy may present an effective treatment for metastatic brain cancer and glioblastoma.^37^ Therefore, eliminating glioma cell stemness is one of the critical strategies for clinical treatment. Some signaling molecules for the maintenance of stemness, such as Hedgehog, CD133, and NF‐κB, have been confirmed as potential therapeutic targets.^38^ Some natural products and their chemical derivatives have been shown to play a role in regulating glioma cell stemness.^39^ Our study suggests that RIP2/NF‐κB is a potential target for treating glioma stemness. This may provide theoretical support for clinical treatment. However, the regulatory mechanism of RIP2/NF‐κB is not fully understood and needs further study.

## 5 CONCLUSION

In conclusion, our study shows that RIP2 is involved in the induction of TMZ resistance in gliomas. Furthermore, the RIP2/NF‐κB signaling pathway induces enhanced stemness and plays an important role in TMZ resistance. Therefore, the combined application of RIP2 or NF‐κB inhibitors and TMZ may be a new strategy for treating RIP2‐related drug‐resistant gliomas.

## AUTHOR CONTRIBUTIONS

All listed authors designed the study, performed the experiments and the statistical analysis, and wrote the manuscript. All authors have read the manuscript and approved the final version.

## FUNDING INFORMATION

This work was supported by the National Natural Science Foundation of China (No. 81241076).

## CONFLICT OF INTEREST

The authors do not have any possible conflicts of interest.

## Supporting information


**Appendix S1:** Supporting information

## Data Availability

The data that support the findings of this study are available in the Supplementary Material of this article.

## References

[1] Ferris SP , Hofmann JW , Solomon DA , Perry A . Characterization of gliomas: from morphology to molecules. Virchows Arch. 2017;471(2):257‐269. 28674742 10.1007/s00428-017-2181-4

[2] Wang H , Xu T , Jiang Y , et al. The challenges and the promise of molecular targeted therapy in malignant gliomas. Neoplasia. 2015;17(3):239‐255. 25810009 10.1016/j.neo.2015.02.002 PMC4372648

[3] Tom MC , Cahill DP , Buckner JC , Dietrich J , Parsons MW , Yu JS . Management for Different Glioma Subtypes: are all low‐grade gliomas created equal? Am Soc Clin Oncol Educ Book. 2019;39:133‐145. 31099638 10.1200/EDBK_238353

[4] Fleischmann DF , Schön R , Corradini S , et al. Multifocal high‐grade glioma radiotherapy safety and efficacy. Radiat Oncol. 2021;16(1):165. 34454558 10.1186/s13014-021-01886-3 PMC8400399

[5] Zhang X , Zhang W , Mao XG , Cao WD , Zhen HN , Hu SJ . Malignant intracranial high grade glioma and current treatment strategy. Curr Cancer Drug Targets. 2019;19(2):101‐108. 29848277 10.2174/1568009618666180530090922

[6] Karachi A , Dastmalchi F , Mitchell DA , Rahman M . Temozolomide for immunomodulation in the treatment of glioblastoma. Neuro Oncol. 2018 Nov 12;20(12):1566‐1572. 29733389 10.1093/neuonc/noy072 PMC6231207

[7] Xia Q , Liu L , Li Y , Zhang P , Han D , Dong L . Therapeutic perspective of temozolomide resistance in glioblastoma treatment. Cancer Invest. 2021;39(8):627‐644. 34254870 10.1080/07357907.2021.1952595

[8] Wu Y , Yao Y , Yun Y , Wang M , Zhu R . MicroRNA‐302c enhances the chemosensitivity of human glioma cells to temozolomide by suppressing P‐gp expression. Biosci Rep. 2019;39(9):BSR20190421. 31409725 10.1042/BSR20190421 PMC6744599

[9] Huang W , Zhong Z , Luo C , et al. The miR‐26a/AP‐2α/Nanog signaling axis mediates stem cell self‐renewal and temozolomide resistance in glioma. Theranostics. 2019;9(19):5497‐5516. 31534499 10.7150/thno.33800 PMC6735392

[10] Wu S , Li X , Gao F , de Groot JF , Koul D , Yung WKA . PARP‐mediated PARylation of MGMT is critical to promote repair of temozolomide‐induced O6‐methylguanine DNA damage in glioblastoma. Neuro Oncol. 2021;23(6):920‐931. 33433610 10.1093/neuonc/noab003 PMC8168825

[11] Zhu D , Tu M , Zeng B , et al. Up‐regulation of miR‐497 confers resistance to temozolomide in human glioma cells by targeting mTOR/Bcl‐2. Cancer Med. 2017;6(2):452‐462. 28064447 10.1002/cam4.987 PMC5313645

[12] Babu D , Mudiraj A , Yadav N , Chandrashekhar YBVK , Panigrahi M , Prakash Babu P . Rabeprazole has efficacy per se and reduces resistance to temozolomide in glioma via EMT inhibition. Cell Oncol (Dordr). 2021;44(4):889‐905. 33948872 10.1007/s13402-021-00609-w PMC12980772

[13] Hofmann SR , Girschick L , Stein R , Schulze F . Immune modulating effects of receptor interacting protein 2 (RIP2) in autoinflammation and immunity. Clin Immunol. 2021;223:108648. 33310070 10.1016/j.clim.2020.108648

[14] Zhou Y , Hu L , Tang W , et al. Hepatic NOD2 promotes hepatocarcinogenesis via a RIP2‐mediated proinflammatory response and a novel nuclear autophagy‐mediated DNA damage mechanism. J Hematol Oncol. 2021;14(1):9. 33413510 10.1186/s13045-020-01028-4 PMC7791875

[15] Singel SM , Batten K , Cornelius C , et al. Receptor‐interacting protein kinase 2 promotes triple‐negative breast cancer cell migration and invasion via activation of nuclear factor‐kappaB and c‐Jun N‐terminal kinase pathways. Breast Cancer Res. 2014;16(2):R28. 24642040 10.1186/bcr3629 PMC4053227

[16] Zhang H , Chin AI . Role of Rip2 in development of tumor‐infiltrating MDSCs and bladder cancer metastasis. PLoS One. 2014;9(4):e94793. 24733360 10.1371/journal.pone.0094793 PMC3986223

[17] Jaafar R , Mnich K , Dolan S , et al. RIP2 enhances cell survival by activation of NF‐ĸB in triple negative breast cancer cells. Biochem Biophys Res Commun. 2018;497(1):115‐121. 29421659 10.1016/j.bbrc.2018.02.034

[18] Wang D , Chen J , Li R , et al. PAX5 interacts with RIP2 to promote NF‐κB activation and drug‐resistance in B‐lymphoproliferative disorders. J Cell Sci. 2016;129(11):2261‐2272. 27122187 10.1242/jcs.183889

[19] Hu YH , Jiao BH , Wang CY , Wu JL . Regulation of temozolomide resistance in glioma cells via the RIP2/NF‐κB/MGMT pathway. CNS Neurosci Ther. 2021;27(5):552‐563. 33460245 10.1111/cns.13591 PMC8025621

[20] Bush NA , Chang SM , Berger MS . Current and future strategies for treatment of glioma. Neurosurg Rev. 2017;40(1):1‐14. 27085859 10.1007/s10143-016-0709-8

[21] Luo C , Xu S , Dai G , Xiao Z , Chen L , Liu Z . Tumor treating fields for high‐grade gliomas. Biomed Pharmacother. 2020;127:110193. 32407989 10.1016/j.biopha.2020.110193

[22] Nassar D , Blanpain C . Cancer stem cells: basic concepts and therapeutic implications. Annu Rev Pathol. 2016;11:47‐76. 27193450 10.1146/annurev-pathol-012615-044438

[23] Batlle E , Clevers H . Cancer stem cells revisited. Nat Med. 2017;23(10):1124‐1134. 28985214 10.1038/nm.4409

[24] Najafi M , Mortezaee K , Majidpoor J . Cancer stem cell (CSC) resistance drivers. Life Sci. 2019;234:116781. 31430455 10.1016/j.lfs.2019.116781

[25] Muftuoglu Y , Pajonk F . Targeting Glioma Stem Cells. Neurosurg Clin N Am. 2021;32(2):283‐289. 33781508 10.1016/j.nec.2021.01.002 PMC8010221

[26] Ludwig K , Kornblum HI . Molecular markers in glioma. J Neurooncol. 2017;134(3):505‐512. 28233083 10.1007/s11060-017-2379-y PMC5568999

[27] Barzegar Behrooz A , Syahir A , Ahmad S . CD133: beyond a cancer stem cell biomarker. J Drug Target. 2019;27(3):257‐269. 29911902 10.1080/1061186X.2018.1479756

[28] Ahmed EM , Bandopadhyay G , Coyle B , Grabowska A . A HIF‐independent, CD133‐mediated mechanism of cisplatin resistance in glioblastoma cells. Cell Oncol (Dordr). 2018;41(3):319‐328. 29492900 10.1007/s13402-018-0374-8 PMC5951876

[29] Song WS , Yang YP , Huang CS , et al. Sox2, a stemness gene, regulates tumor‐initiating and drug‐resistant properties in CD133‐positive glioblastoma stem cells. J Chin Med Assoc. 2016;79(10):538‐545. 27530866 10.1016/j.jcma.2016.03.010

[30] Gong R , Li ZQ , Fu K , Ma C , Wang W , Chen JC . Long noncoding RNA PVT1 promotes stemness and temozolomide resistance through miR‐365/ELF4/SOX2 Axis in glioma. Exp Neurobiol. 2021;30(3):244‐255. 34230224 10.5607/en20060 PMC8278140

[31] Zhang Q , Lenardo MJ , Baltimore D . 30 years of NF‐κB: a blossoming of relevance to human pathobiology. Cell. 2017;168(1–2):37‐57. 28086098 10.1016/j.cell.2016.12.012 PMC5268070

[32] Kunnumakkara AB , Shabnam B , Girisa S , et al. Inflammation, NF‐κB, and chronic diseases: how are they linked? Crit Rev Immunol. 2020;40(1):1‐39. 32421977 10.1615/CritRevImmunol.2020033210

[33] Cai X , Yang Y , Xia W , et al. RIP2 promotes glioma cell growth by regulating TRAF3 and activating the NF‐κB and p38 signaling pathways. Oncol Rep. 2018;39(6):2915‐2923. 29693188 10.3892/or.2018.6397

[34] Teeuwssen M , Fodde R . Wnt signaling in ovarian cancer stemness, EMT, and therapy resistance. J Clin Med. 2019;8(10):1658. 10.3390/jcm8101658 PMC6832489 31614568

[35] Liu L , Tao T , Liu S , et al. An RFC4/Notch1 signaling feedback loop promotes NSCLC metastasis and stemness. Nat Commun. 2021;12(1):2693. 33976158 10.1038/s41467-021-22971-x PMC8113560

[36] Zhu R , Gires O , Zhu L , et al. TSPAN8 promotes cancer cell stemness via activation of sonic hedgehog signaling. Nat Commun. 2019;10(1):2863. 31253779 10.1038/s41467-019-10739-3 PMC6599078

[37] Sadanandan N , Shear A , Brooks B , et al. Treating metastatic brain cancers with stem cells. Front Mol Neurosci. 2021;14:749716. 34899179 10.3389/fnmol.2021.749716 PMC8651876

[38] Alves ALV , Gomes INF , Carloni AC , et al. Role of glioblastoma stem cells in cancer therapeutic resistance: a perspective on antineoplastic agents from natural sources and chemical derivatives. Stem Cell Res Ther. 2021;12(1):206. 33762015 10.1186/s13287-021-02231-x PMC7992331

[39] Wang L , Guo J , Zhou J , Wang D , Kang X , Zhou L . NF‐κB maintains the stemness of colon cancer cells by downregulating miR‐195‐5p/497‐5p and upregulating MCM2. J Exp Clin Cancer Res. 2020;39(1):225. 33109220 10.1186/s13046-020-01704-w PMC7592593

