# Supplementary material for: Mechanism of RIP2 enhancing stemness of glioma cells induces temozolomide resistance
Source: CNS Neurosci Ther. 2022 Oct 2;28(12):2319–30. doi: 10.1111/cns.13981 (PMC9627370; doi:10.1111/cns.13981)
Supplement: Supplementary file 1 — Appendix S1: Supporting information [file CNS-28-2319-s001.pdf]

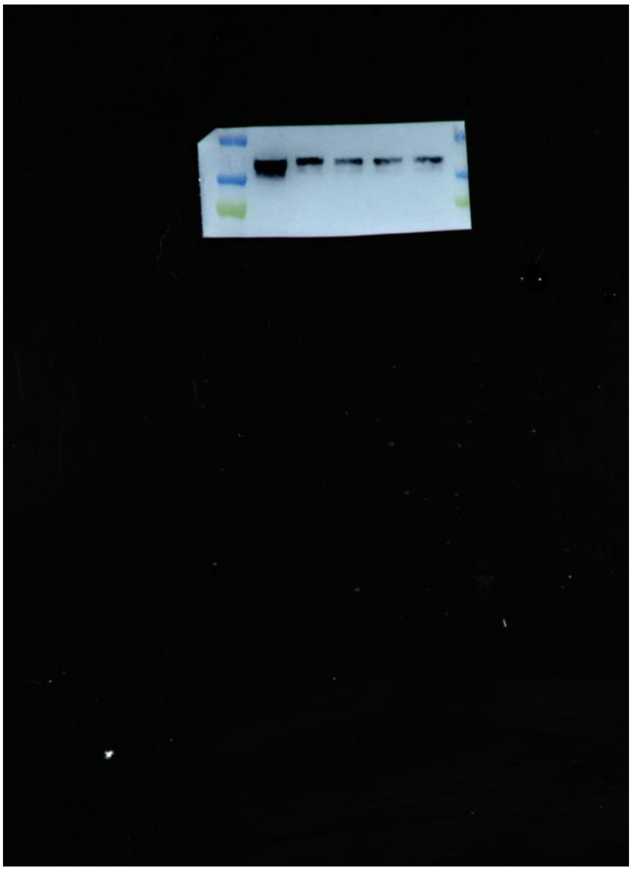

Full unedited blot for Figure 1A RIP2

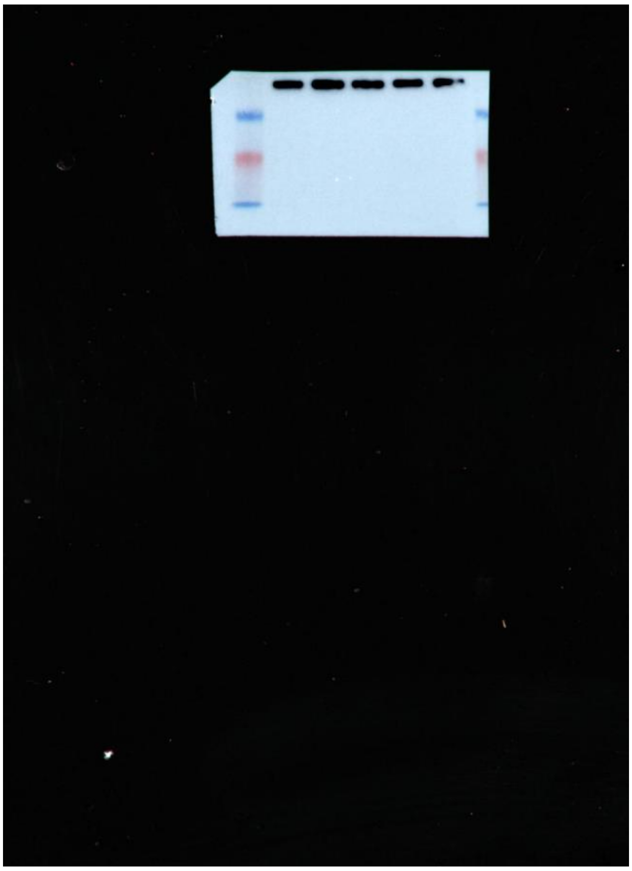

Full unedited blot for Figure 1A GAPDH

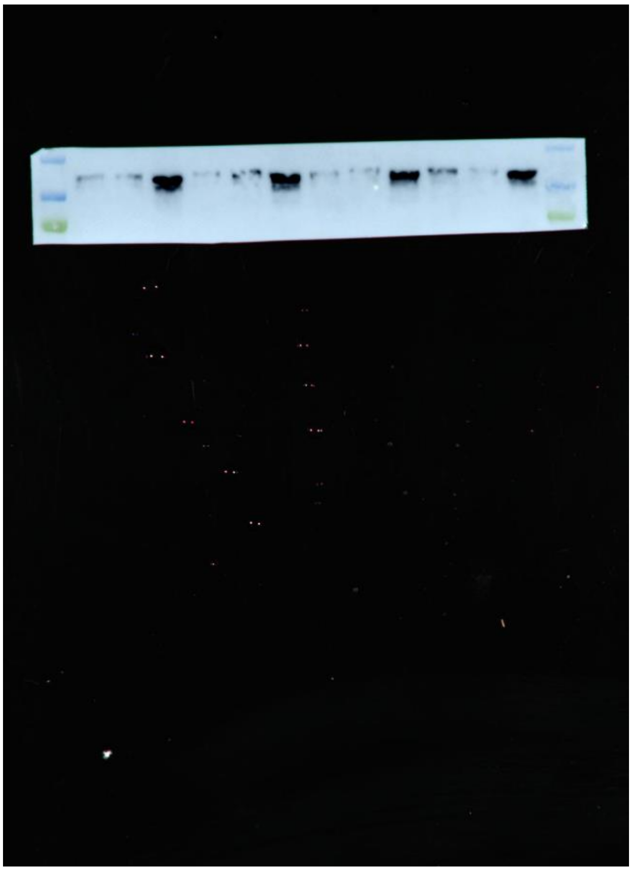

Full unedited blot for Figure 1F RIP2

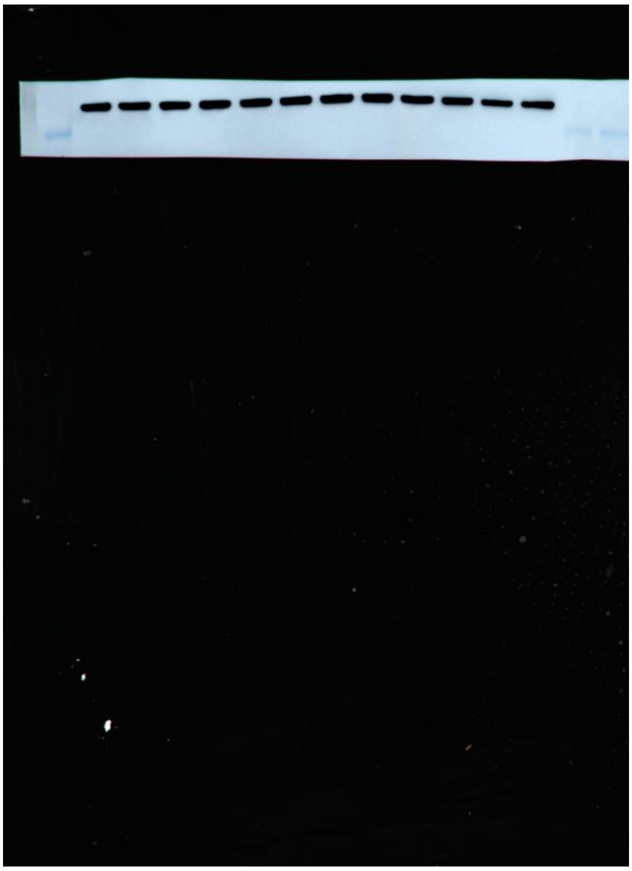

Full unedited blot for Figure 1F GAPDH

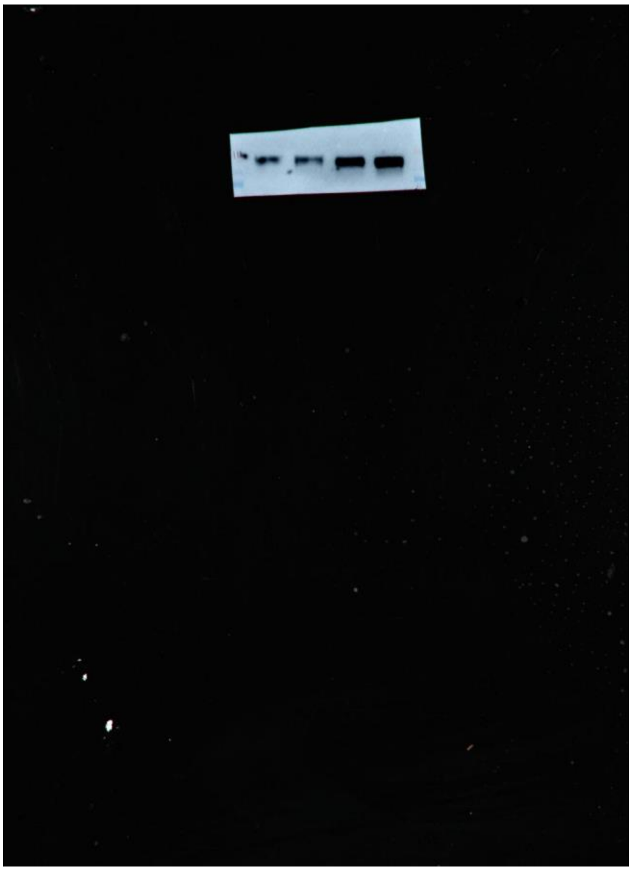

Full unedited blot for Figure 2C CD133

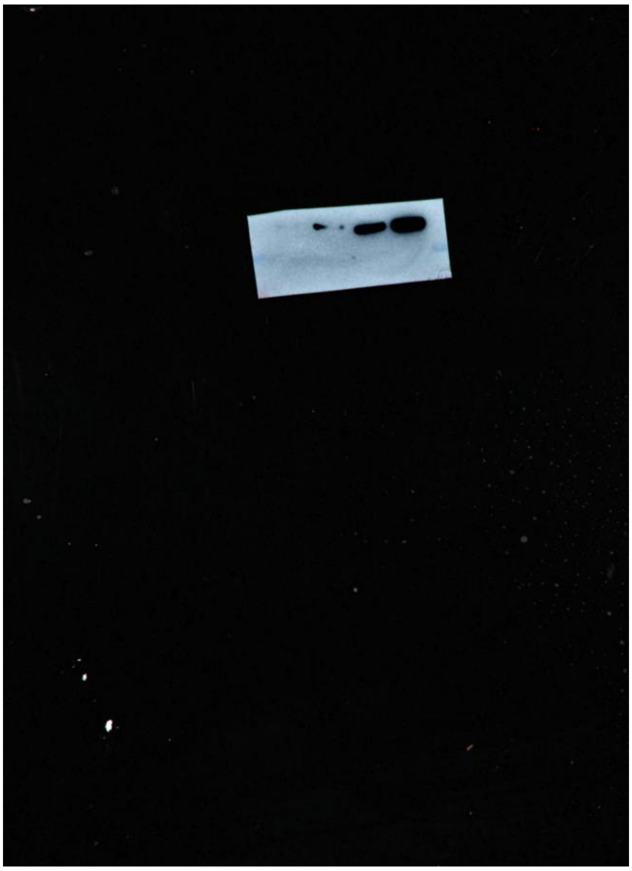

Full unedited blot for Figure 2C SOX2

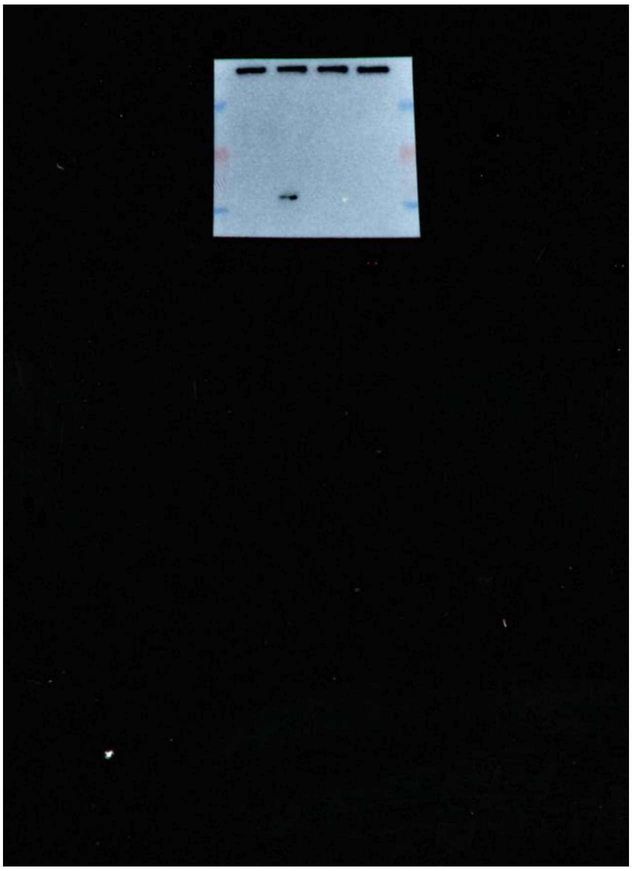

Full unedited blot for Figure 2C GAPDH

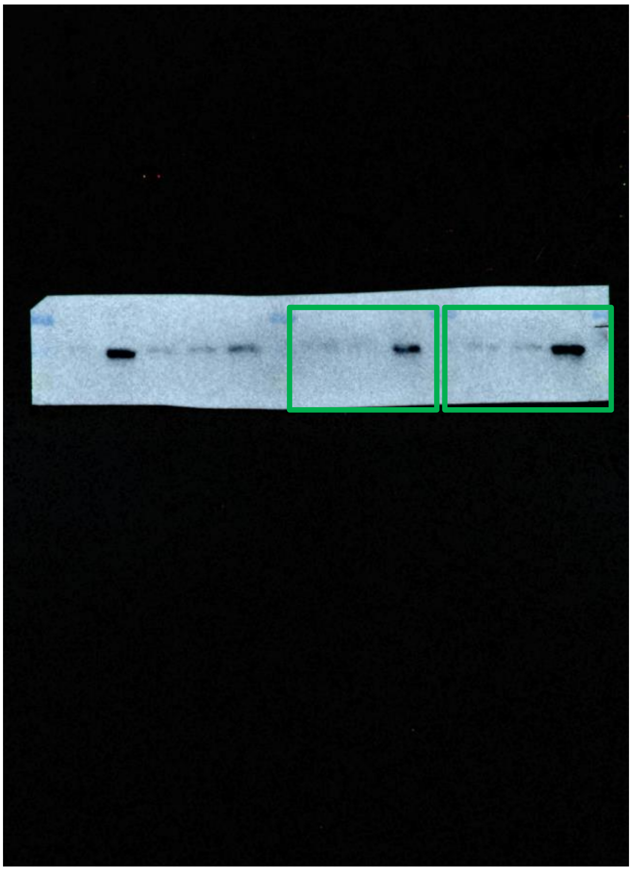

Full unedited blot for Figure 3B RIP2 ； Full unedited blot for Figure 2D RIP2

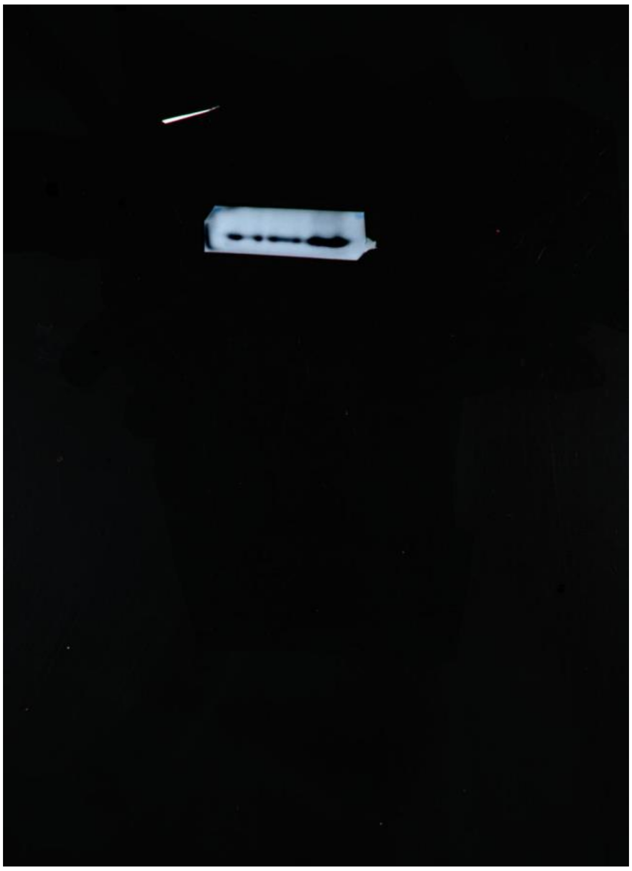

Full unedited blot for Figure 2D CD133

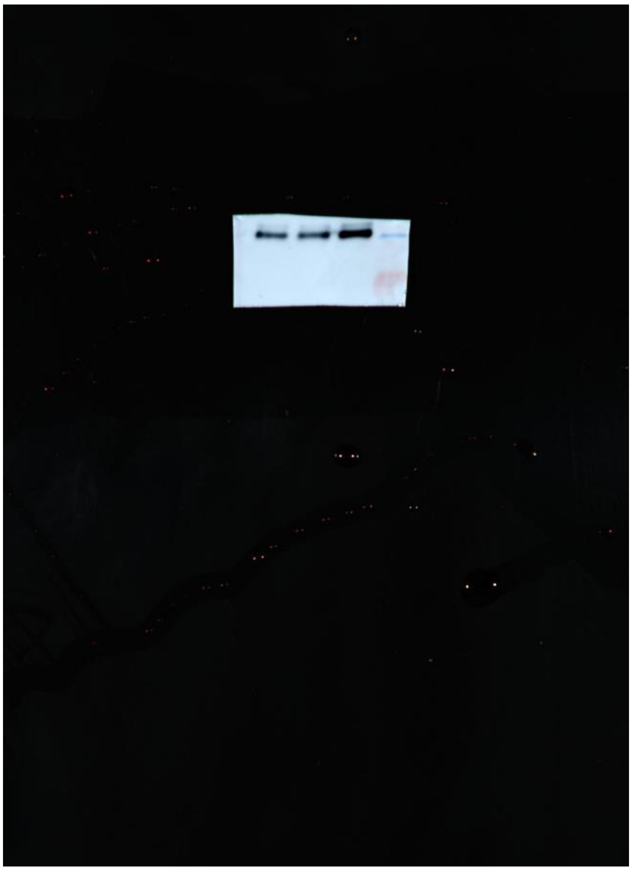

Full unedited blot for Figure 2D SOX-2

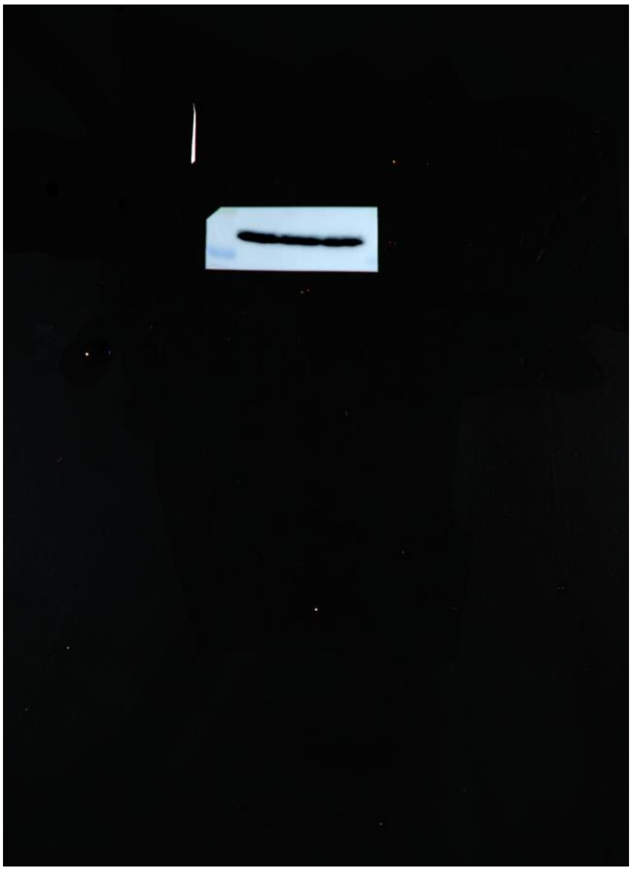

Full unedited blot for Figure 2D GAPDH

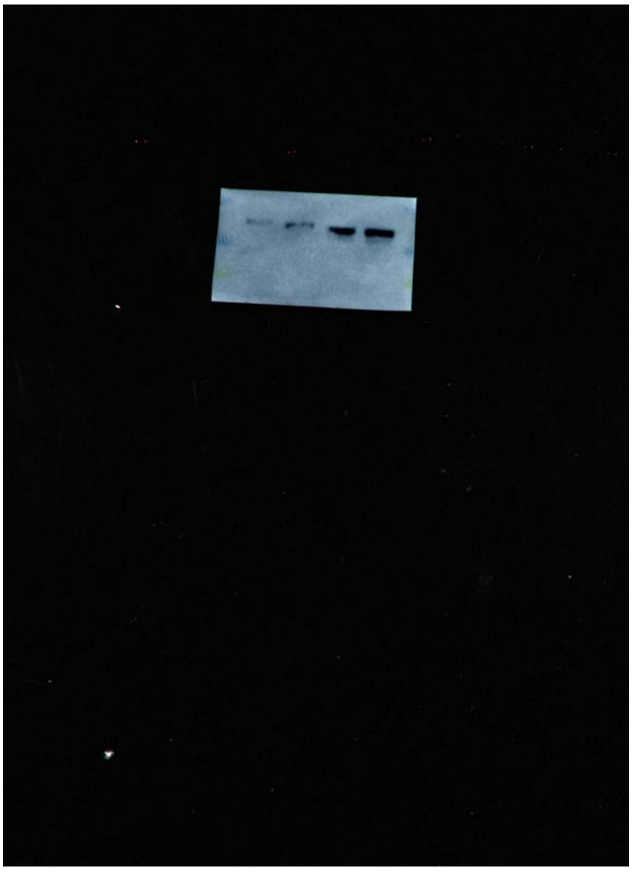

Full unedited blot for Figure 3A p-NF-κB p65

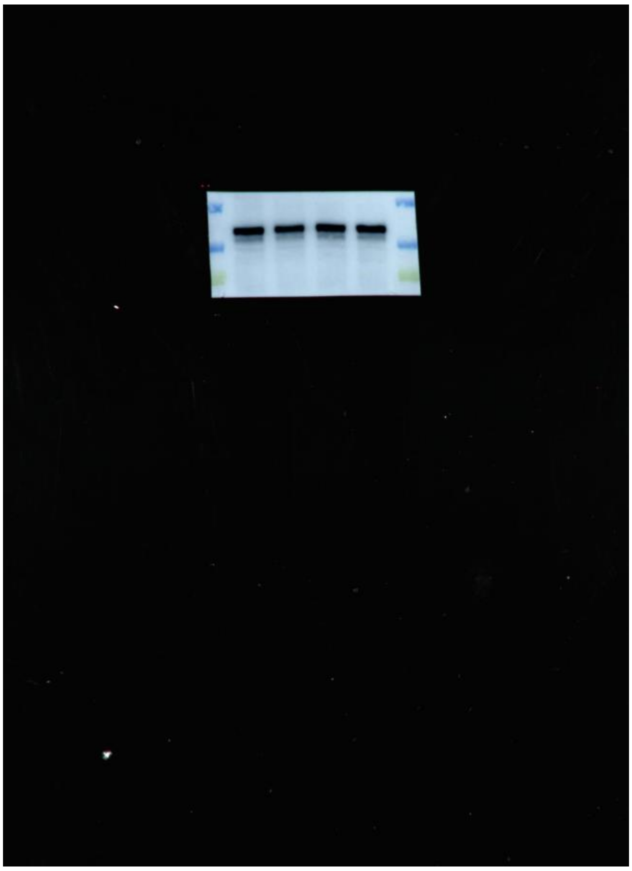

Full unedited blot for Figure 3A NF-κB p65

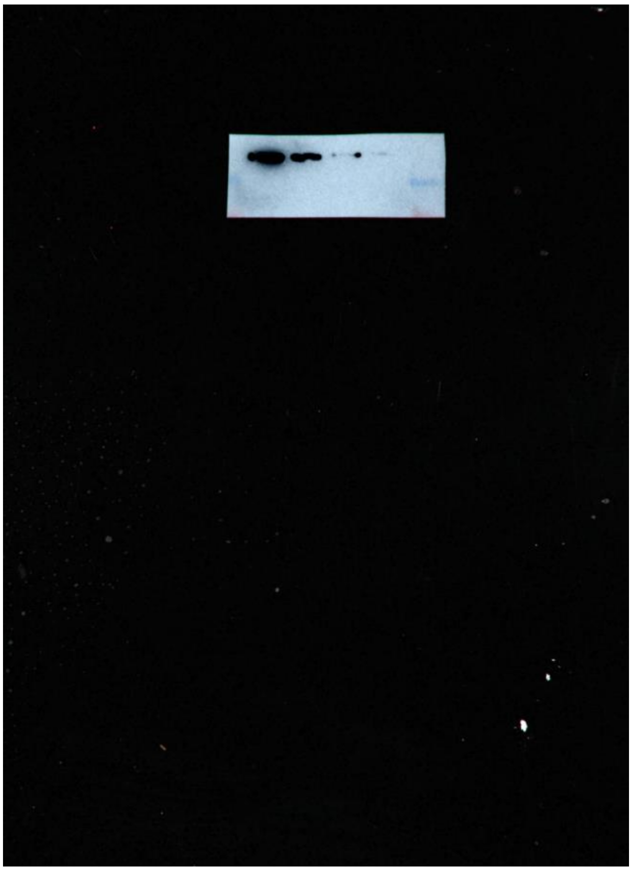

Full unedited blot for Figure 3A IκB α

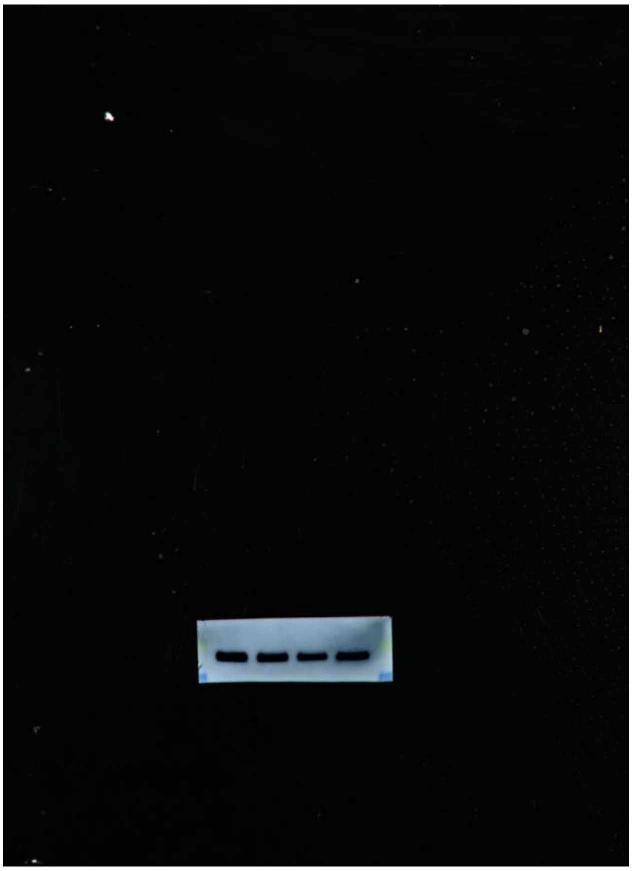

Full unedited blot for Figure 3A GAPDH

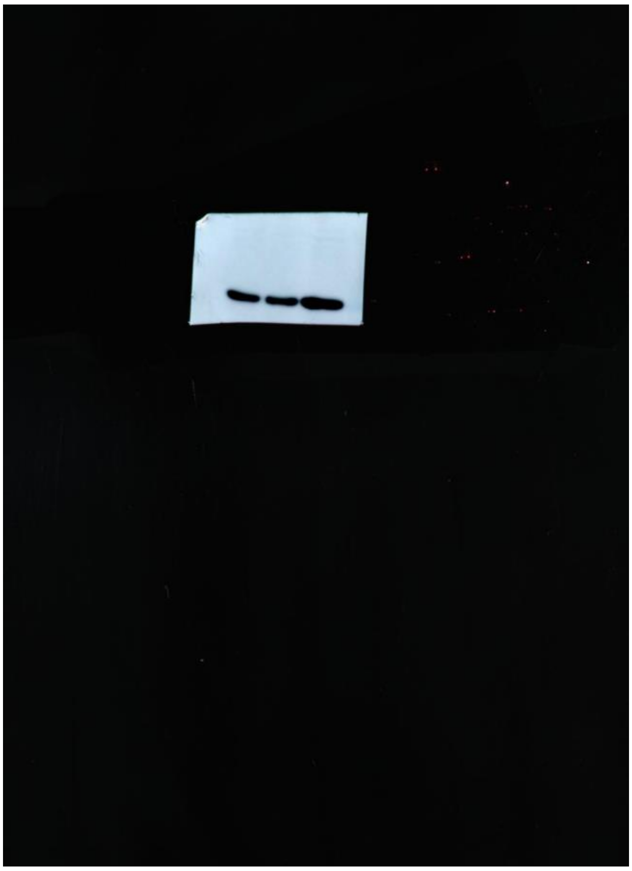

Full unedited blot for Figure 3B p-NF-κB p65

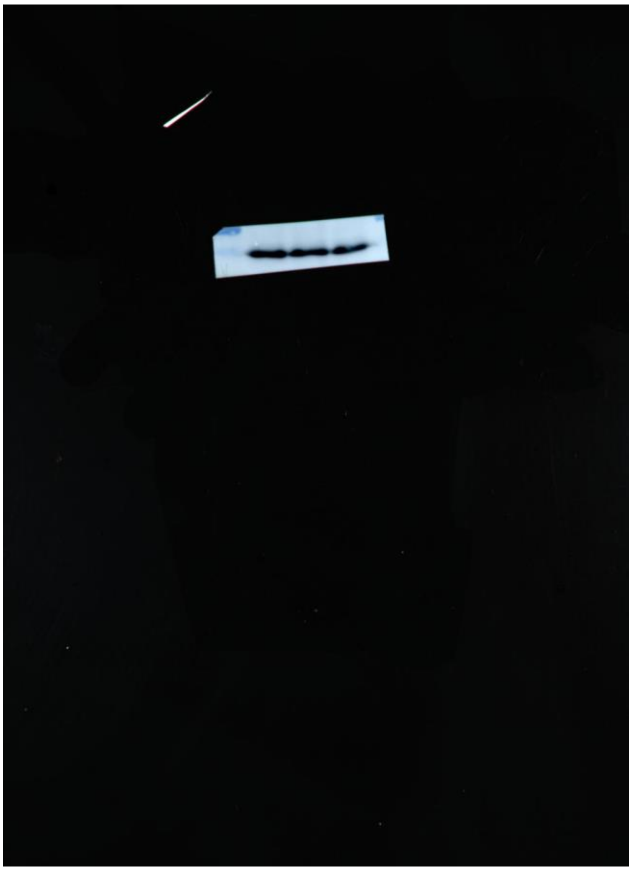

Full unedited blot for Figure 3B NF-κB p65

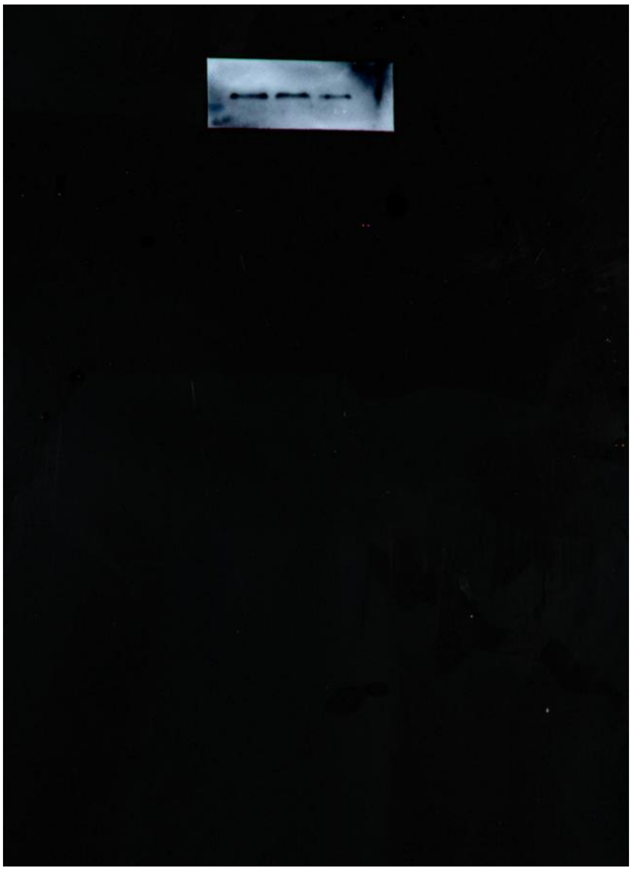

Full unedited blot for Figure 3B IκB α

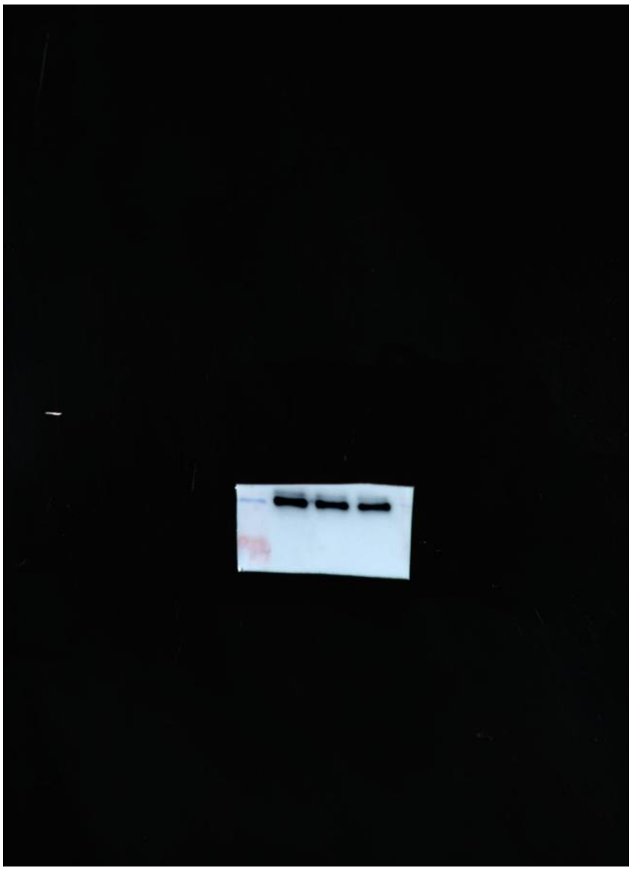

Full unedited blot for Figure 3B GAPDH

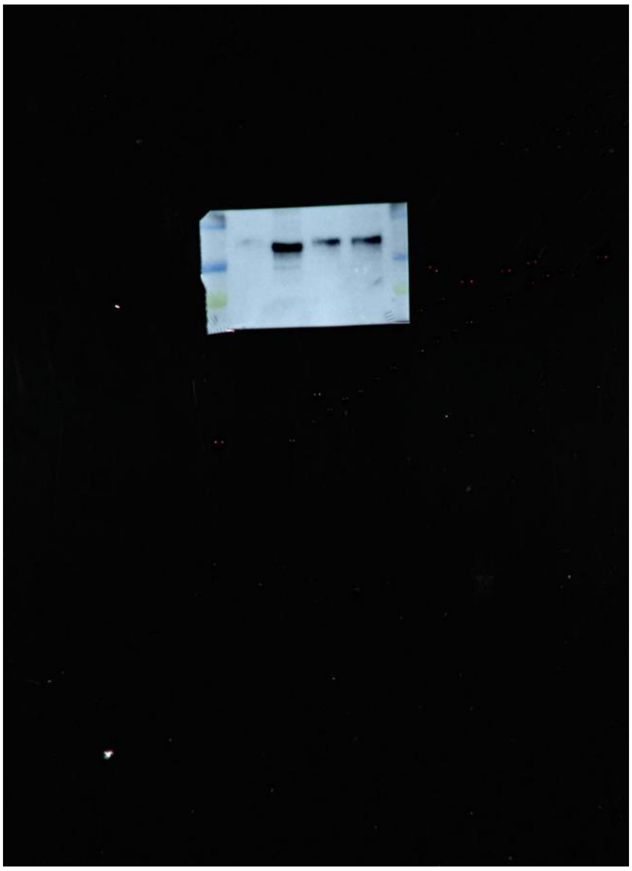

Full unedited blot for Figure 3C p-NF-κB p65

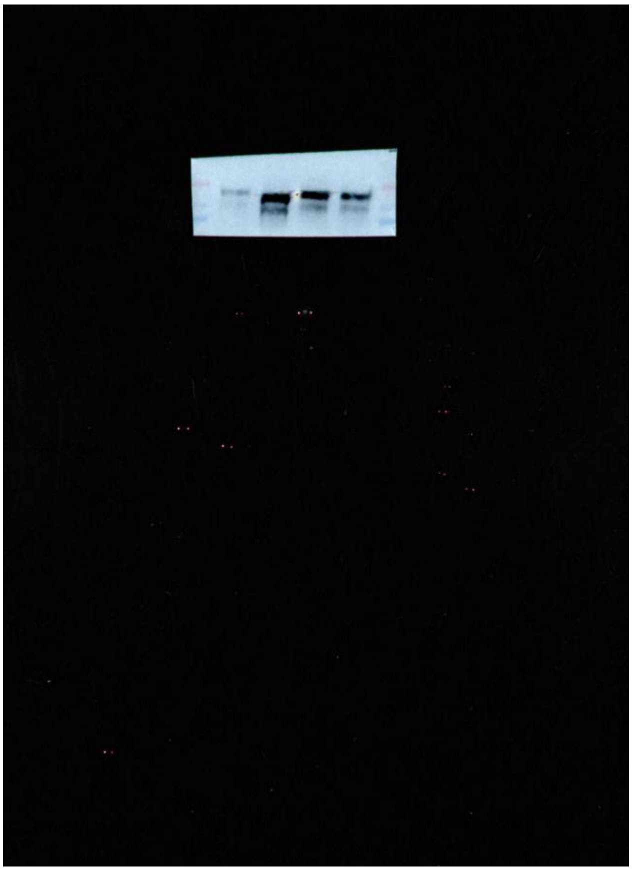

Full unedited blot for Figure 3C CD133

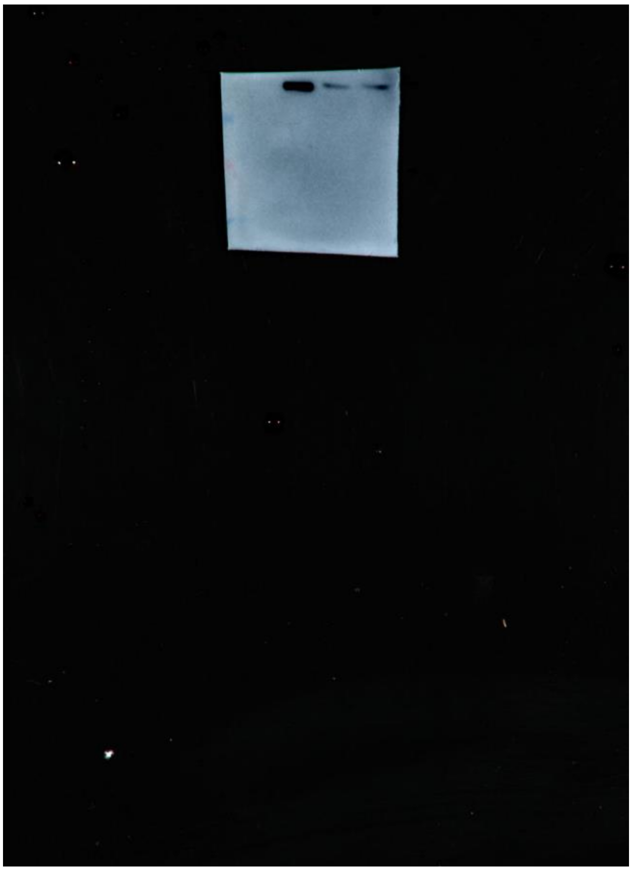

Full unedited blot for Figure 3C SOX-2

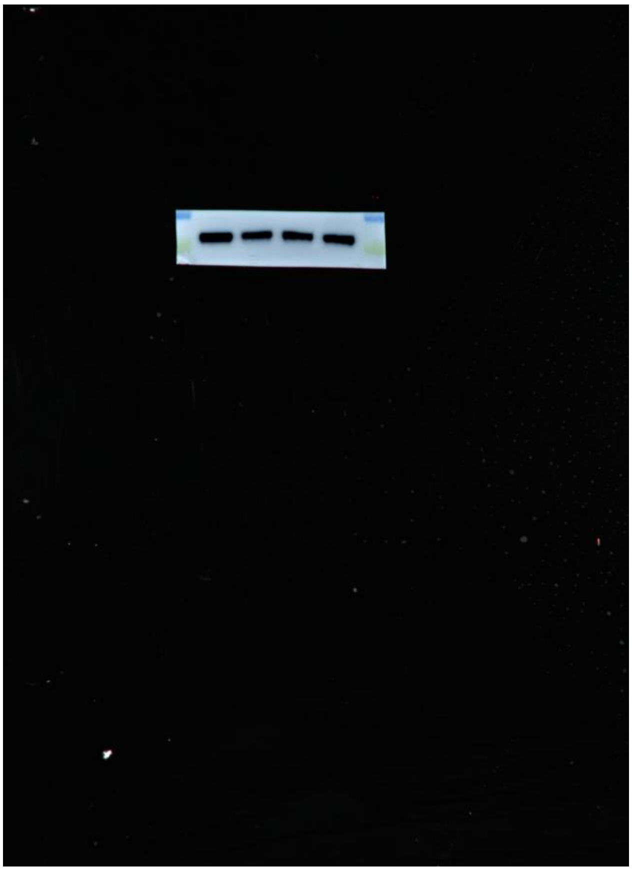

Full unedited blot for Figure 3 GAPDH

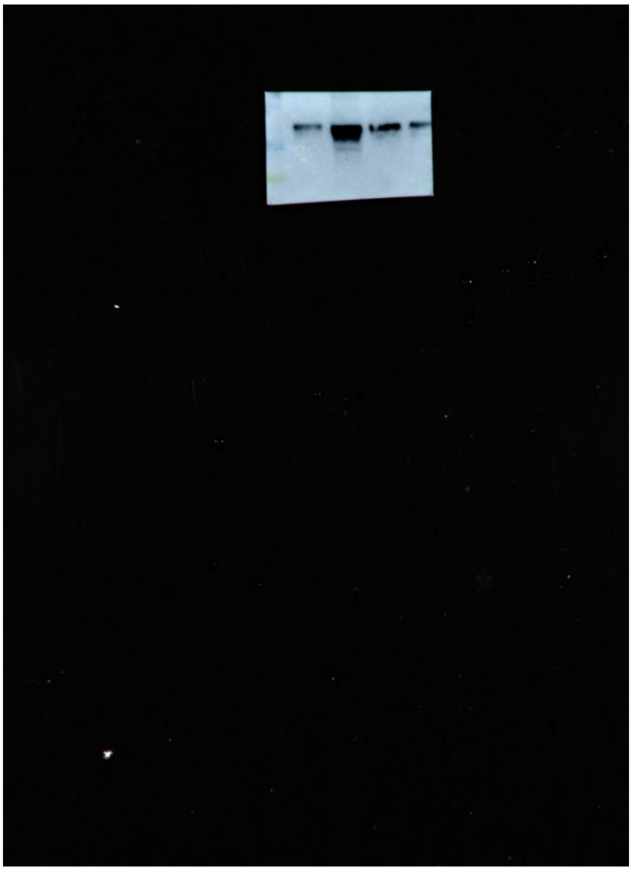

Full unedited blot for Figure 3D p-NF-κB p65

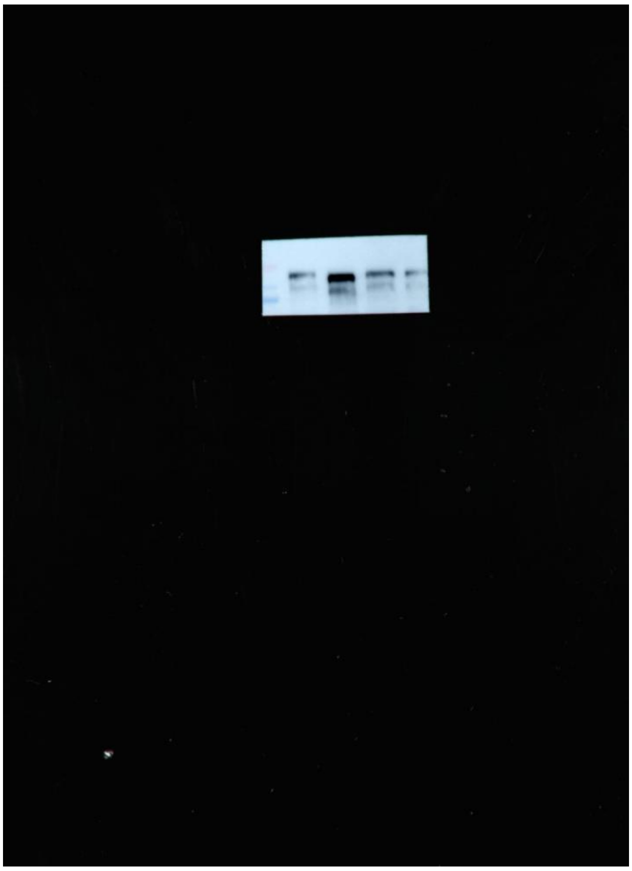

Full unedited blot for Figure 3D CD133

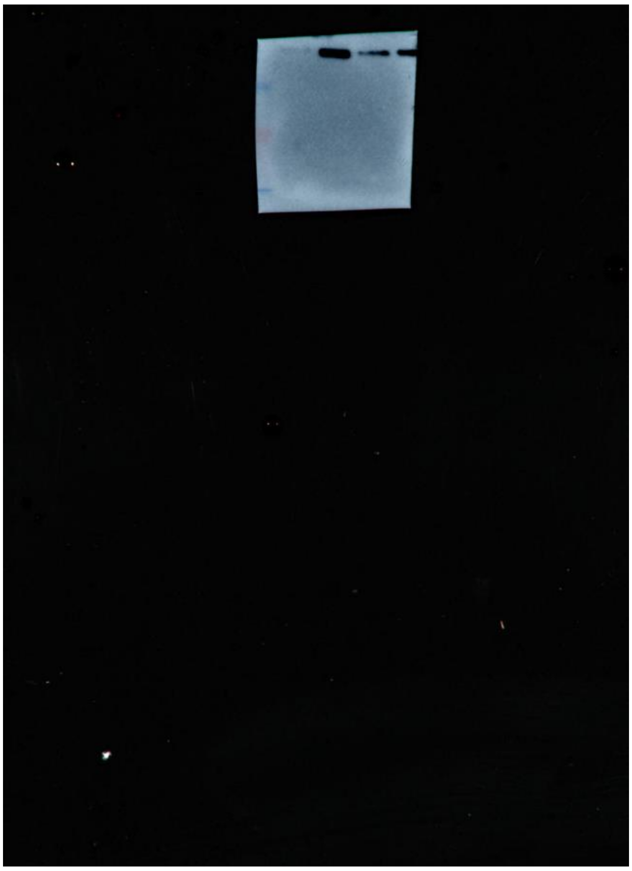

Full unedited blot for Figure 3D SOX-2

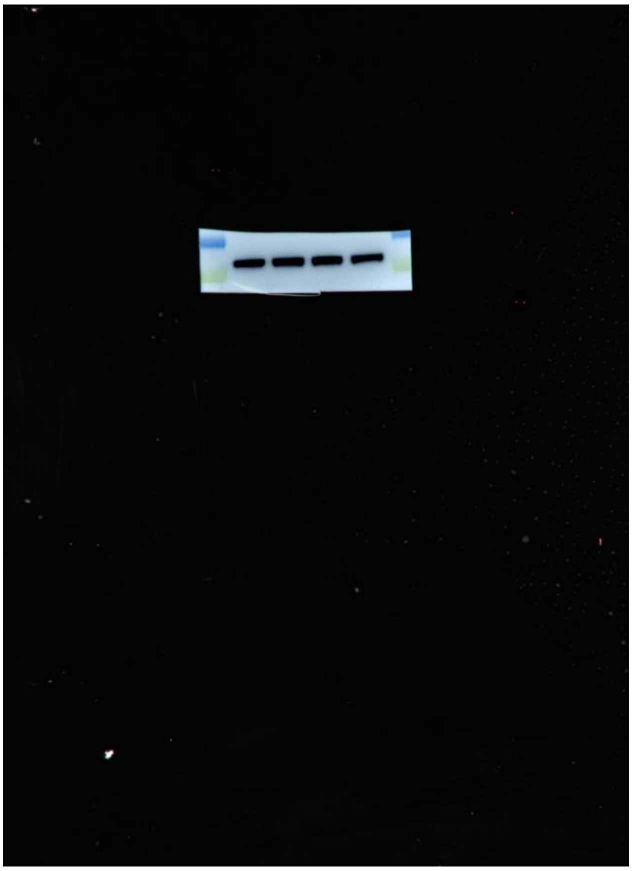

Full unedited blot for Figure 3D GAPDH
